# Gut Microbiota and Metabolic Syndrome: A Narrative Review

**DOI:** 10.3390/biology15141115

**Published:** 2026-07-10

**Authors:** Ioanna Kotsiri, Maria Prokou, Charalampia Melangeli Domazinaki, Eirini Papadakaki, Emmanouil Magiorkinis

**Affiliations:** 1 Second Department of Internal Medicine, Asklepieion General Hospital Voulas, 16673 Athens, Greece; 2 Department of Laboratory Haematology, Metaxa Anticancer Memorial Hospital, 18537 Pireas, Greece

**Keywords:** gut microbiota and obesity, metabolic syndrome, insulin resistance, intestinal microbiota, short-chain fatty acids, probiotics, prebiotics

## Abstract

Obesity is a major global health problem and is closely associated with a broad range of metabolic disorders, including metabolic syndrome (MetS), dyslipidemia, hypertension, atherosclerosis, type 2 diabetes mellitus, and cardiovascular disease. The gut microbiota plays a central role in maintaining intestinal epithelial integrity, regulating glucose and lipid metabolism, and modulating immune function. Through the gut–brain axis, it also contributes to appetite regulation and energy homeostasis by influencing the release of anorexigenic hormones. Dysbiosis, including alterations in the relative abundance of major bacterial phyla such as *Firmicutes* and *Bacteroidetes*, has been associated with increased intestinal permeability, metabolic endotoxemia, and chronic low-grade inflammation, all of which may contribute to the development of obesity and insulin resistance. Diets rich in plant-derived fiber can beneficially shape gut microbiota composition. Bacterial fermentation of dietary fiber produces short-chain fatty acids (SCFAs), including butyrate, acetate, and propionate, which contribute to intestinal barrier integrity, inflammatory regulation, immune regulation, and metabolic homeostasis. Overall, the interaction between gut microbiota, diet, and host metabolic pathways represents a promising field for therapeutic and nutritional interventions aimed at preventing and managing MetS and metabolic diseases.

## Simple Summary

Obesity, a hallmark manifestation of metabolic syndrome, is a major health problem linked to several metabolic diseases. Increasing evidence suggests that the gut microbiota plays an important role in regulating metabolism, immune function, and appetite. Diet, particularly dietary fiber intake, can positively influence gut microbiota composition and promote the production of beneficial metabolites such as short-chain fatty acids. Understanding the interaction between diet, gut microbiota, and obesity may help develop new strategies for the prevention and management of metabolic disorders.

## 1. Introduction

Obesity is a major global public health problem. Since 1990, obesity rates have more than doubled, affecting both adult and pediatric populations. In 2022, more than one billion people worldwide were classified as obese, with a body mass index (BMI) greater than 30 kg/m^2^, while approximately 43% of the global population was classified as overweight [1].

Obesity is particularly prevalent in the Western world and is mainly attributed to dietary changes, including the consumption of high-calorie diets rich in fats and carbohydrates, sedentary lifestyle, and genetic predisposition [2]. It is associated with a wide range of metabolic disorders and comorbidities that can significantly affect overall health. These include metabolic syndrome (MetS); impaired glucose metabolism; insulin resistance; metabolic dysfunction-associated fatty liver disease (MAFLD), formerly referred to as non-alcoholic fatty liver disease (NAFLD); obstructive sleep apnea; depression; fertility disorders; cardiovascular diseases; hypertension; and dyslipidemia, which is characterized by elevated cholesterol levels and reduced high-density lipoprotein (HDL) cholesterol levels [2,3].

Obesity is commonly assessed using body mass index (BMI), with a BMI ≥ 30 kg/m^2^ generally used to define obesity. A BMI of 30–34.9 kg/m^2^ corresponds to class I obesity, 35–39.9 kg/m^2^ to class II obesity, and ≥40 kg/m^2^ to class III obesity [2]. However, BMI does not directly measure body fat or fat distribution; therefore, body fat percentage and indices of central adiposity may provide a more accurate assessment of obesity-related metabolic risk. In recent years, there has been increasing scientific interest in the role of the gut microbiome in the pathophysiology of obesity and MetS, both at diagnostic and therapeutic levels.

The gut microbiota consists of a complex community of microorganisms, including bacteria, fungi, viruses, archaea, and protozoa. Through metabolic, immunological, and neuroendocrine mechanisms, these microorganisms contribute to host metabolic homeostasis [4] and immune regulation while maintaining a symbiotic relationship with the host. Furthermore, the gut microbiota plays an important role in the gut–brain axis, influencing satiety and energy balance [5].

Several factors, including age, unhealthy dietary habits, and geographical location, can alter gut microbiota composition [6]. Such alterations may promote dysbiosis and contribute to the development of immune-mediated and metabolic disorders [4]. Excess adipose tissue, as observed in obesity, activates innate immune responses at the cellular level and promotes a chronic low-grade inflammatory state, which contributes to the pathogenesis of insulin resistance and type 2 diabetes mellitus [6,7].

Through the production of metabolites such as short-chain fatty acids (SCFAs), the gut microbiome can alter intestinal barrier function, trigger inflammatory responses, and contribute to metabolic dysregulation [8].

Moreover, microbial metabolites, such as SCFAs, as well as structural microbial components, including peptidoglycans and lipopolysaccharides (LPSs), can interact with host cell receptors and modulate multiple signaling pathways, producing effects that may be either beneficial or detrimental to host health [4].

## 2. Review Methodology

This narrative review was designed to summarize current knowledge regarding the relationship between gut microbiota, obesity, and MetS, with particular emphasis on microbial metabolites, inflammation, intestinal barrier dysfunction, bile acid metabolism, gut–brain axis signaling, and therapeutic interventions. A literature search was performed using PubMed/MEDLINE, Scopus, and Web of Science. Additional relevant articles were identified through manual screening of reference lists from selected publications.

The search included combinations of the following terms: “gut microbiota”, “gut microbiome”, “intestinal microbiota”, “metabolic syndrome”, “obesity”, “insulin resistance”, “type 2 diabetes”, “short-chain fatty acids”, “SCFAs”, “dysbiosis”, “intestinal permeability”, “metabolic endotoxemia”, “lipopolysaccharide”, “inflammation”, “bile acids”, “gut-brain axis”, “probiotics”, “prebiotics”, “synbiotics”, “fecal microbiota transplantation”, “metformin”, “SGLT2 inhibitors”, “GLP-1 receptor agonists”, and “advanced glycation end products”.

The search focused mainly on articles published in English up to 2026, with priority given to recent original studies, clinical studies, meta-analyses, systematic reviews, and high-quality narrative reviews. Seminal experimental studies were also included when they provided important mechanistic insights into the microbiota–metabolism relationship. Articles were considered eligible if they addressed gut microbiota composition or function in relation to obesity, MetS, insulin resistance, dyslipidemia, hypertension, MAFLD, inflammation, or microbiota-targeted interventions. Studies not directly related to gut microbiota or MetS, articles with insufficient methodological detail, non-English publications when an English version was not available, and duplicate records were excluded. Particular attention was given to recent publications from 2021–2025, including systematic reviews, meta-analyses, randomized controlled trials, and studies using multi-omics approaches, in order to reflect recent advances in microbiome research.

The selected literature was evaluated according to relevance to the scope of the review, study design, population studied, mechanistic contribution, and consistency with existing evidence. Because this is a narrative review, no formal risk-of-bias assessment or quantitative synthesis was performed.

## 3. Definition of MetS (WHO/IDF)

MetS is a cluster of metabolic abnormalities characterized by dyslipidemia, elevated triglyceride levels, low HDL cholesterol, arterial hypertension, abdominal obesity (defined by waist circumference and body mass index (BMI)), impaired glucose tolerance (IGT), and insulin resistance. Its presence is associated with an increased risk of coronary heart disease (CHD), atherosclerotic cardiovascular disease (CVD), and type 2 diabetes mellitus (T2DM) [9].

Different international organizations use slightly different criteria to define MetS. The first formal definition was proposed in 1998 by the World Health Organization (WHO), and since then only minor modifications have been introduced. According to the WHO definition, the main criterion is insulin resistance and at least two of the following: elevated blood pressure, hypertriglyceridemia and/or low HDL cholesterol, obesity, and microalbuminuria [10]. The European Group for the Study of Insulin Resistance (EGIR) also identified insulin resistance as the core diagnostic criterion while excluding microalbuminuria [11]. In contrast, the International Diabetes Federation (IDF) definition emphasizes central obesity plus at least two of the following criteria: elevated triglycerides (TGs) and/or low HDL cholesterol, hypertension, and impaired fasting glucose [12]. The National Cholesterol Education Program Adult Treatment Panel III (NCEP ATP III) defines MetS based on the presence of at least three out of five criteria, without requiring a single mandatory component [13].

**Figure 1:**
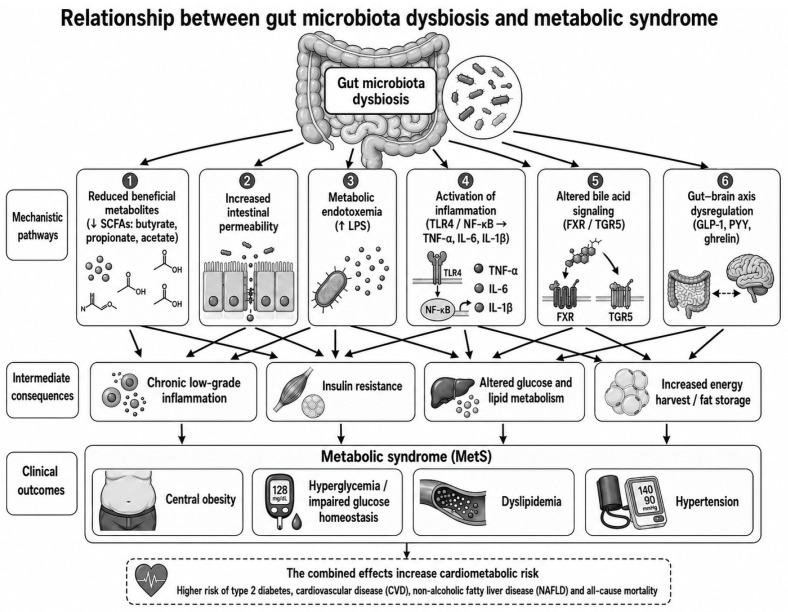
Schematic representation of the major mechanisms linking gut microbiota dysbiosis with MetS. Dysbiosis may reduce beneficial metabolite production, particularly short-chain fatty acids (SCFAs), increase intestinal permeability, promote lipopolysaccharide (LPS) translocation and metabolic endotoxemia, activate inflammatory signaling pathways such as Toll-like receptor 4/nuclear factor-κB (TLR4/NF-κB), alter bile acid signaling through farnesoid X receptor (FXR) and Takeda G protein-coupled receptor 5 (TGR5), and impair gut–brain axis regulation involving glucagon-like peptide-1 (GLP-1), peptide YY (PYY), and ghrelin. These mechanisms contribute to chronic low-grade inflammation, insulin resistance, altered glucose and lipid metabolism, increased energy harvest and fat storage, and the clinical components of MetS, including central obesity, dyslipidemia, hypertension, and impaired glucose homeostasis. MetS, metabolic syndrome; SCFAs, short-chain fatty acids; LPS, lipopolysaccharide; TLR4, Toll-like receptor 4; NF-κB, nuclear factor-κB; FXR, farnesoid X receptor; TGR5, Takeda G protein-coupled receptor 5; GLP-1, glucagon-like peptide-1; PYY, peptide YY.

Although these definitions share several core components, including central obesity or central adiposity, dyslipidemia, hypertension, and impaired glucose metabolism, they differ in diagnostic emphasis and clinical implications [9,10,11,12,13]. The WHO and EGIR definitions prioritize insulin resistance as a central feature, making them more mechanistically oriented but less practical in routine clinical settings, where direct assessment of insulin resistance is not always available [10,11,14,15]. In contrast, the IDF definition emphasizes central obesity as a mandatory criterion, which may increase sensitivity for identifying individuals with abdominal adiposity but may miss metabolically unhealthy individuals without marked central obesity [12,14]. The NCEP ATP III definition is more clinically pragmatic because it does not require one mandatory component and allows diagnosis based on any three of five criteria [13,16]. However, this flexibility may identify a more heterogeneous population. These differences affect estimated MetS prevalence, risk stratification, and comparability across epidemiological and microbiome studies [9,14,17]. Therefore, when interpreting studies linking gut microbiota with MetS, the diagnostic definition used should be considered, because different criteria may select populations with different degrees of insulin resistance, adiposity, inflammation, and cardiometabolic risk [9,14]. Beyond its diagnostic criteria, MetS can be viewed as the clinical consequence of interacting metabolic, inflammatory, and neuroendocrine pathways. As summarized in Figure 1, gut microbiota dysbiosis may contribute to MetS through reduced production of beneficial metabolites such as SCFAs, increased intestinal permeability, metabolic endotoxemia, activation of inflammatory signaling, altered bile acid metabolism, and dysregulation of the gut–brain axis [4,5,8,18,19].

## 4. The Human Microbiome

### 4.1. Composition of the Microbiome

The gut microbiota plays a crucial role in maintaining metabolic homeostasis and host immune function, and its contribution to the development of obesity is increasingly recognized [3]. The composition of the gut microbiota is established during the early stages of life, beginning in the prenatal period, and is influenced by factors such as maternal health, mode of delivery, infant nutrition, and drug exposure. Infants born vaginally have a microbial composition resembling the mother’s vaginal flora, whereas infants delivered by cesarean section tend to exhibit reduced colonization by Bacteroides and Bifidobacterium and increased abundance of skin-associated microorganisms resembling their mother’s skin microbiota. As children grow, their microbiota gradually matures and becomes more similar to that of adults. The human intestinal microbiota includes up to 10^13^–10^14^ symbiotic microorganisms, and their distribution differs along the gastrointestinal tract, with the large intestine hosting the highest microbial density, ranging between 10^11^ and 10^12^ cells/mL [3].

The dominant microbial phyla in the human gastrointestinal tract include Firmicutes, Bacteroidetes, Proteobacteria, Actinobacteria, Fusobacteria and Verrucomicrobia [3,6]. In many studies, Firmicutes and Bacteroidetes are reported as the dominant bacterial phyla in the adult gut microbiota, together accounting for a large proportion of the total bacterial community; however, their relative abundance varies considerably between individuals, populations, dietary patterns, and methodological approaches [20].

Most digestible nutrients, including simple carbohydrates, proteins, and lipids, are absorbed mainly in the small intestine. In contrast, non-digestible dietary components, particularly dietary fibers, resistant starches, prebiotics, glucans, and endogenous proteins, reach the colon, where they serve as substrates for microbial fermentation [20,21]. Resistant starch, for example, escapes digestion in the small intestine and is fermented in the large intestine, leading to the production of microbial metabolites such as SCFAs [22,23]. Therefore, although many nutrients are absorbed before reaching the colon, overall dietary patterns strongly influence gut microbiota composition by determining the type and amount of fermentable substrates available to colonic microorganisms [20].

### 4.2. Microbiome Composition Changes in Obesity and MetS

The composition of the intestinal microbiota is strongly influenced by dietary patterns. Alterations in microbiome composition can lead to dysbiosis, which has been associated with chronic low-grade inflammation and the development of metabolic disorders, including increased appetite and alterations in lipid synthesis and storage, ultimately contributing to obesity [3,24].

These alterations may increase the production of microbial metabolites that promote lipogenesis, such as acetate, which can serve as a substrate for hepatic acetyl-CoA production and de novo lipogenesis, and trimethylamine N-oxide (TMAO), which has been linked to altered lipid metabolism, steatohepatitis, and cardiovascular risk. In addition, pro-inflammatory pathways may be activated through increased intestinal permeability and enhanced translocation of bacterial products, particularly lipopolysaccharide (LPS), into the systemic circulation [5]. LPS can activate Toll-like receptor 4 (TLR4)-mediated signaling, leading to nuclear-factor-κB (NF-κB) activation and increased production of inflammatory cytokines such as TNF-α, IL-6, and IL-1β [25], thereby contributing to metabolic endotoxemia, insulin resistance, and chronic low-grade inflammation [18,26]. These processes represent key mechanisms linking gut microbiota impairment with MetS. This inflammatory state may further promote adipose tissue dysfunction, hepatic lipid accumulation, endothelial dysfunction, hypertension, dyslipidemia, and impaired glucose homeostasis, thereby contributing to the major clinical components of MetS [19].

Advanced glycation end products (AGEs) may represent an additional link between gut microbiota impairment and MetS. AGEs are formed through non-enzymatic glycation and oxidation of proteins, lipids, and nucleic acids and may originate endogenously, particularly under hyperglycemic and oxidative conditions, or exogenously through the diet, especially after consumption of highly processed foods or foods cooked at high temperatures [27,28]. Dietary AGEs can interact with the gut microbiota and have been associated with changes in microbial composition, reduced intestinal barrier integrity, and increased intestinal inflammation [27,29,30]. At the host level, AGEs can bind to the receptor for advanced glycation end products (RAGE), activating oxidative stress and inflammatory signaling pathways, including NF-κB-mediated cytokine production [27,31]. These mechanisms may contribute to insulin resistance, adipose tissue inflammation, dyslipidemia, endothelial dysfunction, hepatic steatosis, and cardiovascular risk, thereby linking AGEs with several components of MetS [28,32]. Conversely, dysbiosis and impaired metabolic control may further favor AGE accumulation, suggesting a bidirectional relationship between AGEs, gut microbiota, and MetS-related inflammation [27,33].

In the intestines of healthy individuals, Firmicutes are usually present in high proportions, followed by Bacteroidetes. The Firmicutes/Bacteroidetes ratio has been associated with the production of SCFAs, including acetic, propionic, and butyric acids, which are essential for maintaining metabolic homeostasis. Disturbances in this ratio, particularly a relative decrease in Bacteroidetes and an increase in Firmicutes, have been associated with an increased risk of obesity [34].

Although gut microbiota dysbiosis has also been associated with non-metabolic disorders such as celiac disease, colorectal cancer, inflammatory bowel disease, and neurological conditions, these topics are beyond the main scope of the present review [35,36]. Therefore, the following sections focus specifically on microbiota-related mechanisms relevant to MetS, including altered microbial metabolites, intestinal permeability, low-grade inflammation, bile acid metabolism, gut–brain axis regulation, insulin resistance, dyslipidemia, hypertension, and MAFLD.

In one study of patients with metabolic dysfunction-associated fatty liver disease (MAFLD), differences in gut microbiota composition and microbial metabolic activity were reported according to disease severity [37]. Specifically, mild/moderate MAFLD was associated with enrichment of bacterial genera such as *Blautia* sp. and *Eubacterium* sp., whereas more advanced fibrosis was associated with enrichment of *Prevotella* spp. and *Bacteroides* spp. In the same study, mild/moderate MAFLD was associated with increased microbial enzymatic activity related to lactate, acetate, and formate metabolism, whereas advanced fibrosis was associated with increased activity of enzymes involved in butyrate, D-lactate, and propionate metabolism [37]. However, because these findings are derived from a single study, they should be interpreted with caution and should not be generalized to all patients with MAFLD. Further studies in larger, independent, and ethnically diverse cohorts are required to confirm these observations and clarify whether these microbiome signatures are consistently associated with MAFLD severity.

Several studies have investigated differences in gut microbiota composition between individuals with obesity and lean controls. One of the early and frequently cited studies by Ley et al. reported that obesity was associated with a higher relative abundance of Firmicutes and a lower relative abundance of Bacteroidetes, while weight loss was accompanied by an increase in the relative abundance of Bacteroidetes [38,39]. This study was important because it suggested that gut microbiota composition may vary according to host adiposity and metabolic status. However, later studies have not consistently reproduced the same Firmicutes/Bacteroidetes pattern. These discrepancies may reflect differences in diet, age, geography, ethnicity, sequencing methods, sample size, medication exposure, and metabolic phenotype. Therefore, the Firmicutes/Bacteroidetes ratio should be interpreted cautiously and should not be considered a universal marker of obesity or MetS.

The study by Ridaura et al. [40] is among the most important studies supporting a causal relationship between the microbiome and obesity. The study demonstrated that transfer of microbiota from obese individuals and their lean twins to germ-free mice affects host metabolism. Mice that received microbiota from obese individuals showed increased body weight and fat mass, whereas mice that received microbiota from lean individuals remained lean, despite both groups being fed the same diet. These findings support the concept that modulation of the gut microbiota may represent a therapeutic target for metabolic diseases [40]. Gut bacteria can break down complex carbohydrates derived from the intestinal mucosa or from foods such as pectin and sorbitol, which are widely found in fruits and vegetables. They can also metabolize other carbohydrates, including mannose, fructose, cellulose, and sucrose; these carbohydrates are not absorbed in the small intestine but are fermented by colonic bacteria, leading to SCFA production and providing an energy source for the host [38].

In a study by Fei and Zhao, the bacterium *Enterobacter cloacae* was shown to potentially contribute to obesity and insulin resistance when introduced into germ-free mice, likely through endotoxin production and systemic inflammation. A diet rich in plant fibers reduced the abundance of this bacterium and contributed to significant weight loss and improved metabolism [41]. Andoh et al. investigated whether alterations in the intestinal microbiota are associated with obesity by comparing the intestinal microbiota of 10 obese and 10 lean Japanese individuals using 16S *rRNA* gene sequencing. Lean participants had significantly higher microbial diversity than obese participants. Obese subjects had increased Firmicutes and Fusobacteria, whereas Bacteroides was more abundant in lean subjects. No significant differences were observed in the Bacteroidetes/Firmicutes ratio between the two groups, highlighting inconsistencies in the literature. Specifically, the anti-inflammatory species *Faecalibacterium prausnitzii* was increased in lean subjects, whereas pro-inflammatory taxa were more prevalent in obese individuals [42].

As discussed above, early studies suggested an association between obesity and changes in the Firmicutes/Bacteroidetes ratio, although later findings have been inconsistent. Similar findings were reported by Turnbaugh et al., in which reduced levels of Bacteroidetes were detected in cecal samples from obese leptin-deficient mice compared with lean mice [43].

Similar changes in gut microbiota composition have been observed in obese children compared with lean children. Several studies have reported an increase in *Firmicutes* and a relative decrease in Bacteroidetes, leading to an increased Firmicutes/Bacteroidetes ratio. Beneficial taxa such as Bifidobacterium and Lactobacillus were also reduced in obese children, whereas Bacteroides and Sanguibacteroides were increased in lean children. These changes may disrupt intestinal barrier integrity and immune function and may increase the chronic inflammation characteristic of obesity [44,45].

However, these findings should be interpreted with caution. Although an increased Firmicutes/Bacteroidetes ratio has frequently been reported in obesity, this pattern is not universal, and some studies have failed to detect significant differences between obese and lean individuals. Several factors may explain these discrepancies, including differences in age, ethnicity, dietary habits, geographic background, medication exposure, metabolic status, sample size, stool collection protocols, sequencing platforms, and bioinformatic analysis pipelines. In addition, obesity and MetS are heterogeneous conditions, and microbiota alterations may depend more on insulin resistance, diet quality, inflammation, visceral adiposity, or glycemic status than on BMI alone. The Firmicutes/Bacteroidetes ratio is also a broad phylum-level marker that does not capture species- or strain-level differences, microbial gene content, or metabolite production. Therefore, rather than being considered a reliable standalone biomarker of obesity or MetS, this ratio should be interpreted as one of several possible indicators of dysbiosis. Functional characteristics of the microbiome, including SCFA production, bile acid metabolism, endotoxin-related inflammatory signaling, and TMA/TMAO production, may be more informative for understanding the contribution of gut microbiota to MetS.

Taken together, these findings suggest that microbiome composition changes in obesity and MetS should be interpreted not only through broad taxonomic shifts, such as the *Firmicutes*/*Bacteroidetes* ratio, but also through functional pathways involving microbial metabolites, intestinal permeability, inflammatory signaling, bile acid transformation, and host metabolic phenotype.

## 5. Microbiome MetS and Energy Homeostasis

### Short-Chain Fatty Acids: Production, Signaling, and Metabolic Effects

Short-chain fatty acids (SCFAs) are saturated fatty acids containing one to six carbon atoms and represent key microbial metabolites linking diet, gut microbiota, and host metabolism [34,46]. Complex carbohydrates that are not digested in the small intestine, including dietary fibers, resistant starches, prebiotics, glucans, and other fermentable substrates, reach the colon and are metabolized by gut microbiota through fermentation. This process leads mainly to the production of acetate, propionate, and butyrate [4]. The production of SCFAs depends primarily on the fermentation of dietary fibers, with soluble fibers generally being more efficiently fermented than insoluble fibers. Prebiotics such as fructo-oligosaccharides (FOSs), galacto-oligosaccharides (GOSs), lactulose, inulin, and oligofructose can promote SCFA production. Inulin and oligofructose primarily produce acetate, whereas protein fermentation yields branched-chain fatty acids, which may have less favorable metabolic effects [46].

The main SCFAs are acetate, propionate, and butyrate. Acetate, the most abundant systemic SCFA, and propionate are produced mainly by bacterial genera within the Bacteroidetes phylum, particularly Bacteroides spp., as well as by certain Actinobacteria, such as Bifidobacterium spp. Butyrate-producing bacteria are mainly members of the Firmicutes phylum, including Eubacterium, Roseburia, and other taxa belonging to clostridial clusters IV, XIVa, and XVI [4,16,21,25]. Butyrate is synthesized through the butyrate kinase or butyryl-coenzyme A (CoA) pathways, whereas acetate is mainly produced through acetyl-CoA pathways [34].

**Figure 2:**
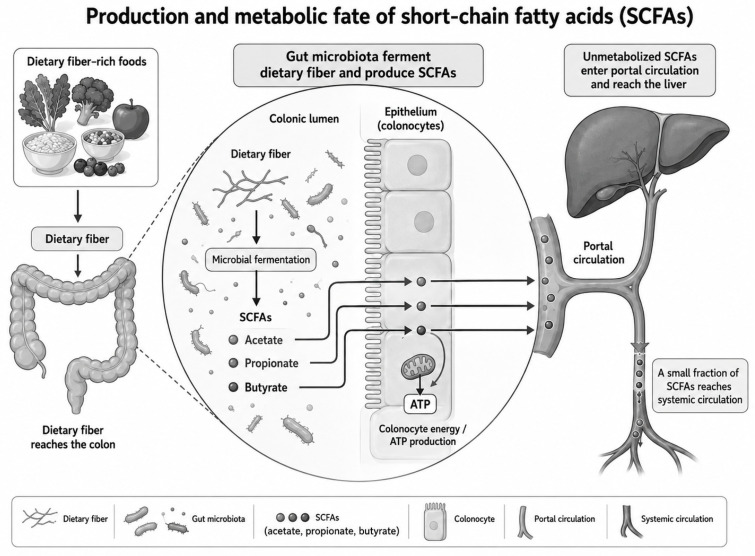
Schematic illustration of SCFA production and distribution. Dietary fiber and other non-digestible carbohydrates reach the colon, where gut microbiota ferment these substrates within the colonic lumen. This process leads mainly to the production of acetate, propionate, and butyrate. Butyrate is used primarily by colonocytes as an energy source and contributes to adenosine triphosphate (ATP) production, mucosal integrity, and maintenance of the intestinal barrier. Acetate and propionate, together with unmetabolized butyrate, may cross the intestinal epithelium and enter the portal circulation, reaching the liver, where they participate in glucose and lipid metabolism. A smaller fraction of SCFAs reaches the systemic circulation and may influence peripheral tissues through metabolic, immune, and neuroendocrine signaling pathways. Abbreviations: SCFAs, short-chain fatty acids; ATP, adenosine triphosphate.

Butyrate is an important energy source for the intestinal epithelium. In enterocytes and colonocytes, SCFAs are metabolized through the tricarboxylic acid (TCA) cycle, contributing to adenosine triphosphate (ATP) production [43]. Butyrate strengthens the intestinal barrier and increases mucin expression through regulation of the MUC2 gene. It also promotes the differentiation of regulatory T lymphocytes (Tregs), participates in the regulation of inflammation, and appears to protect against obesity and insulin resistance [47,48]. Furthermore, butyrate promotes proliferation of normal intestinal epithelial cells, whereas in transformed cells, it may induce differentiation and apoptosis [48]. A portion of SCFAs is used locally by colonocytes, whereas unmetabolized SCFAs enter the portal circulation and reach the liver, with a smaller fraction reaching the systemic circulation, as summarized in Figure 2.

**Figure 3:**
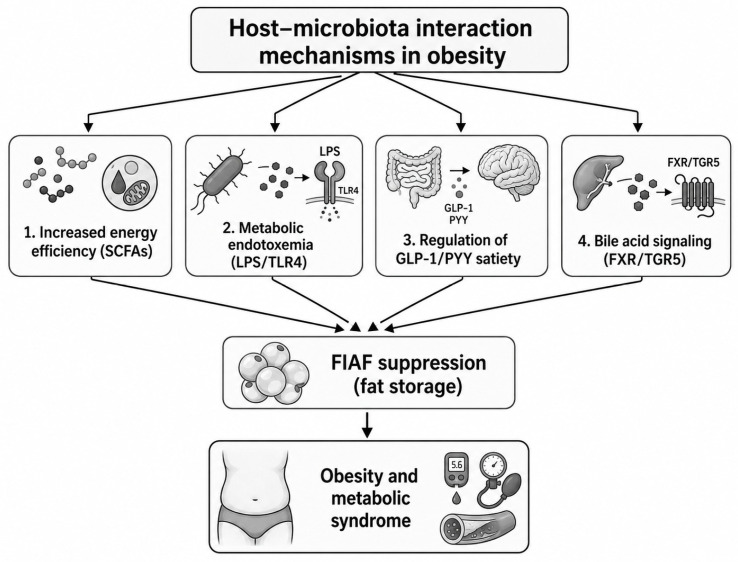
Schematic overview of major host–microbiota pathways involved in obesity and MetS. Gut microbiota may influence host metabolism through increased energy efficiency mediated by short-chain fatty acids (SCFAs), metabolic endotoxemia involving lipopolysaccharide/Toll-like receptor 4 (LPS/TLR4) signaling, regulation of glucagon-like peptide-1/peptide YY (GLP-1/PYY)-mediated satiety, and bile acid signaling through farnesoid X receptor/Takeda G protein-coupled receptor 5 (FXR/TGR5). These pathways may converge on suppression of fasting-induced adipocyte factor (FIAF), also known as angiopoietin-like protein 4 (ANGPTL4), promoting lipoprotein lipase activity, fat storage, obesity, and MetS. SCFAs, short-chain fatty acids; LPS, lipopolysaccharide; TLR4, Toll-like receptor 4; GLP-1, glucagon-like peptide-1; PYY, peptide YY; FXR, farnesoid X receptor; TGR5, Takeda G protein-coupled receptor 5; FIAF, fasting-induced adipocyte factor.

SCFAs also act as signaling molecules through G protein-coupled receptors (GPCRs), including free fatty acid receptor 2 (FFAR2/GPR43), free fatty acid receptor 3 (FFAR3/GPR41), and GPR109A, also known as hydroxycarboxylic acid receptor 2 (HCAR2). These receptors are expressed in intestinal cells and in several peripheral tissues, including immune and neural cells [43]. GPR43 and GPR41 are expressed on the apical membrane of intestinal L-cells, particularly in the distal ileum and colon, where SCFA concentrations are highest [46]. Their activation by SCFAs promotes the secretion of glucagon-like peptide-1 (GLP-1) and peptide YY (PYY), which act on the gut–brain axis, regulate glucose metabolism, and promote satiety [46,47]. Specifically, activation of GPR41 increases PYY release, thereby reducing intestinal motility and enhancing satiety. In contrast, activation of GPR43 has been associated with anti-inflammatory effects and increased secretion of GLP-1 and GLP-2, with downstream effects on insulin, leptin, and ghrelin secretion. Through these mechanisms, SCFAs contribute to appetite regulation and metabolic homeostasis [49]. These host–microbiota interaction mechanisms are summarized in Figure 3.

At the metabolic level, SCFAs influence glucose and lipid metabolism in several tissues. In the liver, SCFAs modulate glucose metabolism by reducing gluconeogenesis and increasing glycogen synthesis [47]. Propionate enters the portal circulation, promotes intestinal gluconeogenesis, and has been associated with reduced hepatic lipogenesis and improved glycemic control [5,48]. Acetate enters the systemic circulation and may be used for energy production through the Krebs cycle. In the hypothalamus, acetate suppresses orexigenic neuropeptide expression, leading to reduced appetite and food intake [50]. Acetate has also been shown to reduce the influx of free fatty acids (FFAs) to the liver, thereby improving insulin sensitivity [48]. In skeletal muscle and adipose tissue, SCFAs activate AMP-activated protein kinase (AMPK), increase glucose transporter type 4 (GLUT4) expression, and enhance glucose uptake. In skeletal muscle, SCFAs also increase glycogen synthesis [47].

SCFAs exert important immunomodulatory effects. They can reduce the secretion of pro-inflammatory cytokines such as tumor necrosis factor-alpha (TNF-α) and interleukin-6 (IL-6) while promoting production of anti-inflammatory cytokines such as interleukin-10 (IL-10). Butyrate has potent anti-inflammatory effects, partly through increased IL-10 production. SCFAs also participate in leukocyte chemotaxis and immune cell migration to sites of inflammation. These anti-inflammatory effects are mediated through the activation of GPCRs such as FFAR2 and FFAR3, as well as through their activity as histone deacetylase (HDAC) inhibitors, which regulate gene expression and immune responses [46]. Propionic acid has been shown to inhibit the mRNA expression of interleukin-1β (IL-1β), IL-6, and TNF-α, as well as the phosphorylation of signal transducer and activator of transcription 3 (STAT3). It also reduces myeloperoxidase levels and induces antioxidant enzyme activity, including superoxide dismutase and catalase, in the colon [47]. These mechanisms are relevant to metabolic syndrome (MetS), in which chronic low-grade inflammation contributes to insulin resistance, adipose tissue dysfunction, endothelial dysfunction, dyslipidemia, hypertension, and metabolic dysfunction-associated fatty liver disease (MAFLD).

However, the metabolic effects of SCFAs are context-dependent. Although SCFAs generally support intestinal barrier integrity, immune regulation, and metabolic homeostasis, acetate may also serve as a substrate for hepatic acetyl-CoA production and de novo lipogenesis under specific nutritional or metabolic conditions. In the study by Zhao et al. [51], liver-specific ATP citrate lyase knockout mice (LAKO mice), in which the gene encoding ATP citrate lyase (ACLY/Acly) was deleted in the liver, did not show reduced de novo lipogenesis (DNL) compared with wild-type mice, either under normal dietary conditions or after consumption of a high-fructose diet. Instead, increased expression of acyl-CoA synthetase short-chain family member 2 (ACSS2), the enzyme that converts acetate to acetyl-CoA, was observed, indicating activation of an alternative microbiome-related metabolic pathway. Furthermore, inhibition of ACSS2 led to a significant reduction in the conversion of fructose to fatty acids in the liver [51], highlighting the role of this pathway in microbiota-associated lipogenesis.

Dietary fat quality may also influence gut microbiota composition, SCFA production, and MetS-related outcomes. Diet strongly influences gut microbiota composition, and several microbial metabolites have been linked to insulin secretion and sensitivity, as well as to the incidence of type 2 diabetes [52]. Diets rich in long-chain fatty acids (LCFAs) have been shown to alter gut microbiota composition and increase the Firmicutes/Bacteroidetes ratio, as well as the abundance of Actinobacteria. LCFAs are efficiently stored in adipose tissue and contribute to fat accumulation. Medium-chain fatty acids (MCFAs) increase energy expenditure, induce satiety, reduce appetite, and may enhance insulin sensitivity in muscle and adipose tissue. By contrast, consumption of monounsaturated fatty acids (MUFAs) has been associated with beneficial metabolic effects and a reduced risk of MetS, partly through modulation of lipid metabolism and apolipoprotein regulation. Polyunsaturated fatty acids (PUFAs), particularly omega-3 PUFAs, have been associated with reductions in adipose tissue fat accumulation, insulin resistance, inflammation, hypertension, atherosclerosis, obesity, cardiovascular disease, and type 2 diabetes mellitus [34]. Thus, SCFA-related pathways should be interpreted within the broader dietary context, including fiber intake, fat quality, and overall dietary pattern.

## 6. Factors Influencing Microbiome Composition

### 6.1. Drugs

Various drugs, including metformin, antibiotics, proton pump inhibitors and laxatives, interact with the gut microbiota and can alter its composition.

#### 6.1.1. Metformin

Several human studies provide evidence that metformin treatment is associated with changes in gut microbiota composition and function. In a large metagenomic analysis, Forslund et al. showed that metformin treatment represents an important confounding factor in type 2 diabetes microbiome studies and is associated with specific changes in gut microbial composition and functional potential [53]. In a clinical study of treatment-naïve patients with type 2 diabetes, Wu et al. reported that metformin treatment improved glycemic parameters and altered the gut microbiome, including changes in microbial pathways related to SCFA metabolism and enrichment of bacterial taxa potentially involved in glucose regulation [54]. Other clinical studies have also reported that metformin use is associated with an increased abundance of *Akkermansia muciniphila* and several SCFA-producing bacteria, suggesting that microbiota modulation may contribute to improved glucose metabolism and inflammatory status [55,56]. However, these findings should be interpreted cautiously, because many studies are observational or have limited sample sizes, and the extent to which microbiota changes directly mediate the glucose-lowering effect of metformin in humans remains incompletely established.

#### 6.1.2. SGLT2 Inhibitors and GLP-1 Receptor Agonists

SGLT2 inhibitors, including empagliflozin and dapagliflozin, have been reported to influence gut microbiota composition and function [57,58]. Available evidence suggests that these agents may reshape the gut microbial ecosystem, increase the abundance of SCFA-producing bacteria, reduce potentially harmful bacterial taxa, improve intestinal barrier integrity, reduce endotoxemia-related inflammation, and modulate bile acid metabolism [57,58,59]. These effects may contribute to improved metabolic and inflammatory profiles, although the exact mechanisms remain under investigation [57,60].

GLP-1 receptor agonists (GLP-1RAs), such as liraglutide, semaglutide, and exenatide, may also interact with the gut microbiota [61,62]. Studies have reported changes in microbial composition, richness, and diversity during GLP-1RA treatment, with potential increases in beneficial bacterial genera involved in metabolic regulation, including *Akkermansia muciniphila* in some experimental studies [61,63,64]. Because gut-derived microbial metabolites, including SCFAs and secondary bile acids, can stimulate enteroendocrine L cells and influence GLP-1 secretion, the relationship between GLP-1 signaling and gut microbiota appears to be bidirectional [58,62].

Other commonly used drugs in patients with obesity, diabetes, and MetS, including statins, antihypertensive agents, antibiotics, and proton pump inhibitors, may also affect the gut microbiota [57,60]. Therefore, medication exposure should be considered when interpreting microbiome studies in MetS.

### 6.2. Bile Acid Metabolism

The gut microbiota participates in bile acid metabolism and plays a key role in host metabolic regulation. Through enzymatic transformations, gut bacteria convert primary bile acids into secondary bile acids, which can influence host physiology. These mechanisms may explain, at least in part, the hypoglycemic effects of metformin through regulation of incretin secretion, hepatic glucose production, and pancreatic β-cell function. Bile acids are cholesterol-derived metabolites that facilitate the digestion and absorption of dietary lipids. They also contribute to metabolic regulation by modulating lipid biosynthesis, glucose metabolism, and immune responses through interactions with receptors such as the farnesoid X receptor (FXR) and Takeda G protein-coupled receptor 5 (TGR5) [65,66].

### 6.3. Gut–Brain Axis

The gut–brain axis is a bidirectional communication system that includes the central nervous system, enteric nervous system, hypothalamic–pituitary–adrenal (HPA) axis, and the intestinal microbiota [8]. Information transmission through this network regulates energy balance and food intake. This balance is achieved through the interplay of satiety and hunger signals, involving the secretion of anorexigenic hormones such as peptide YY (PYY), glucagon-like peptide-1 (GLP-1), and glucose-dependent insulinotropic polypeptide (GIP), which promote satiety, as well as orexigenic hormones such as ghrelin, which stimulate appetite [5,8,67].

The study by Borgmann et al. showed that sensory neurons innervating the gut transmit satiety signals to the brain and regulate glycemic homeostasis. Dysfunction of these neurons has been associated with hyperphagia and weight gain [68]. Through the gut–brain axis, the gut microbiota can influence both neurodevelopmental processes and brain function. Disturbances in communication along this axis have been linked to metabolic diseases, as well as psychiatric and other comorbidities [8], which are often associated with alterations in gut microbiota composition and function.

The vagus nerve plays a crucial role in the gut–brain axis [68]. Vagal sensory neurons carry axons that innervate visceral organs and project to the brainstem [69]. In obesity, dysregulation of vagal signaling has been observed, partly as a result of chronic overnutrition, contributing to insulin resistance. Activation of vagal afferents expressing the glucagon-like peptide 1 receptor (GLP-1R) improves glycemic control, whereas their inhibition has been shown to increase blood glucose levels [68]. Food intake leads to gastric distension, which, together with vagal signaling, contributes to the perception of satiety during a meal [69,70].

Afferent fibers of the vagus nerve regulate food intake through the release of hormones, such as cholecystokinin (CCK), peptide YY (PYY), and glucagon-like peptide 1 (GLP-1), which are secreted by intestinal enteroendocrine cells in response to nutrient intake [68,71].

## 7. Therapeutic Interventions

Microbiome-targeted interventions represent a promising therapeutic approach for MetS because they may act simultaneously on several interconnected mechanisms, including SCFA production, intestinal barrier integrity, metabolic endotoxemia, bile acid metabolism, gut–brain axis signaling, inflammation, and host energy metabolism [72,73]. These interventions include dietary modification, prebiotics, probiotics, synbiotics, postbiotics, fecal microbiota transplantation (FMT), next-generation probiotics, and personalized nutrition strategies [72,74,75,76]. However, their clinical effects remain heterogeneous and are influenced by baseline microbiota composition, dietary background, medication exposure, metabolic phenotype, intervention duration, and host-specific factors [74,75,76].

### 7.1. Nutritional Interventions

Western lifestyle patterns and diets rich in saturated and trans fatty acids, as well as refined sugars, are typically characterized by reduced dietary fiber intake. These factors can alter the composition and function of the gut microbiota, reduce beneficial microbial populations, and contribute to the development of metabolic and cardiovascular diseases. In addition, Western-style diets and high-temperature cooking methods may increase dietary AGE intake, which has been associated with gut microbiota alterations, intestinal inflammation, oxidative stress, and insulin resistance; therefore, reducing the consumption of highly processed and AGE-rich foods may represent an additional nutritional approach for improving metabolic and inflammatory status [27].

**Figure 4:**
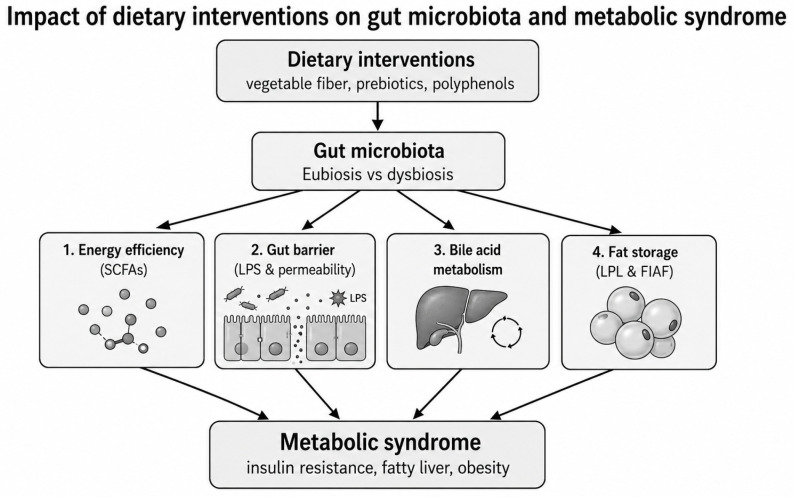
Schematic representation of how dietary interventions may modulate gut microbiota and MetS-related pathways. Vegetable fiber, prebiotics, and polyphenols may shift gut microbiota composition toward eubiosis and influence energy efficiency through short-chain fatty acids (SCFAs), gut barrier function through lipopolysaccharide (LPS)-related permeability pathways, bile acid metabolism, and adipose tissue fat storage through lipoprotein lipase (LPL) and fasting-induced adipocyte factor (FIAF/ANGPTL4). These pathways are linked to key MetS components, including insulin resistance, fatty liver, obesity, dyslipidemia, hypertension, and impaired glucose metabolism. SCFAs, short-chain fatty acids; LPS, lipopolysaccharide; LPL, lipoprotein lipase; FIAF, fasting-induced adipocyte factor; MetS, metabolic syndrome.

Dietary fibers are carbohydrate polymers and may be classified according to their main food source, chemical structure, water solubility, viscosity, and fermentability by the gut microbiota (Figure 4). Based on solubility, they are classified as soluble or insoluble fibers. Insoluble fibers, such as cellulose and hemicellulose, primarily contribute to stool bulking and intestinal motility, whereas soluble fibers, such as β-glucan and pectin, are more readily fermented by gut bacteria and thereby contribute to SCFA production [77,78].

Individuals who follow diets rich in plant-derived fibers, such as the Mediterranean diet, often show an increased abundance of bacterial taxa involved in complex carbohydrate fermentation, including Bacteroides, Prevotella, and Roseburia. These taxa contribute to the production of short-chain fatty acids (SCFAs), particularly butyrate, which supports intestinal barrier integrity, immune regulation, and metabolic homeostasis [79,80].

In a study by De Filippo et al., the role of diet in shaping intestinal microbiota composition was investigated by comparing children from Africa and Italy. Children from Africa, who followed a diet high in plant fibers, showed an increased abundance of Bacteroidetes, mainly genera such as Prevotella and Xylanibacter, and a decreased abundance of Firmicutes. In contrast, children from Italy, who followed a diet rich in fats and proteins, showed an increased abundance of Firmicutes and Proteobacteria [81].

These findings are consistent with the study by Igudesman et al. in adolescents with type 1 diabetes, where increased fiber and carbohydrate intake was associated with higher SCFA concentrations, mainly acetate, as well as an increased abundance of beneficial SCFA-producing microbes such as Roseburia and Ruminococcus gnavus. In contrast, fructose intake was negatively associated with the genus Akkermansia [82].

Overall, diet, and especially fiber intake, plays an important role in shaping the microbiome and regulating metabolism.

### 7.2. Probiotics and Prebiotics

Probiotics and prebiotics can modify intestinal microbiota composition and exert beneficial effects on the host. Probiotics are defined as live microorganisms, including bacteria or fungi, that are ingested through fermented foods or supplements and, when consumed in adequate amounts, confer a health benefit on the host [83].

Prebiotics, in contrast, are usually non-digestible, fermentable fibers such as fructo-oligosaccharides, inulin, oligofructose, galacto-oligosaccharides, and resistant starch, which promote the growth of beneficial bacteria such as Bifidobacterium and Lactobacillus. Synbiotics combine probiotics and prebiotics with the aim of synergistically enhancing their effects [46,84,85].

Most available evidence derives from animal models; however, the existing literature is promising. Recent findings suggest that strain-specific probiotics can exert beneficial effects on obesity-related outcomes in rodents fed a high-fat-diet. Furthermore, Lactobacillus strains have been implicated in reduced body fat and improved lipid and glucose profiles in animal models.

Specifically, *Lactobacillus gasseri* has been associated with weight loss in overweight humans and reduced abdominal adiposity through mechanisms involving lipoprotein lipase inhibition and fatty acid oxidation. Additional mechanisms may include strengthening of the gut barrier, leading to reduced endotoxemia and bacterial translocation [8]. Probiotics may also stimulate the production of anorexigenic hormones from enteroendocrine cells and increase plasma levels of PYY and GLP-1 [5].

Meta-analyses have shown that probiotic administration may have beneficial effects on fasting glucose, insulin resistance, glycated hemoglobin (HbA1c), and lipid metabolism [86,87]. Recent meta-analyses further support the potential role of probiotics and synbiotics in MetS. For example, probiotic or synbiotic supplementation has been associated with improvements in BMI, fasting blood glucose, and LDL cholesterol in patients with MetS. These findings suggest that microbiota-targeted supplementation may improve selected metabolic parameters; however, heterogeneity in probiotic strains, dose, duration, and study populations limits direct comparison between studies [74].

Clinical evidence suggests that probiotic effects are strain-specific and outcome-specific [88]. Preparations containing Lactobacillus and Bifidobacterium species have been most frequently investigated in obesity, insulin resistance, type 2 diabetes, and MetS-related outcomes [74,88]. Some trials and meta-analyses have reported improvements in BMI, waist circumference, fasting glucose, insulin resistance, inflammatory markers, and lipid parameters; however, results are not uniform [74,89,90,91]. Differences in probiotic strain, viability, dose, intervention duration, baseline metabolic status, diet, medication exposure, and host microbiota composition may explain part of this heterogeneity [88,91,92]. Therefore, probiotics should not be considered a uniform intervention, and evidence should be interpreted at the strain or formulation level rather than at the genus level [88,92].

**Figure 5:**
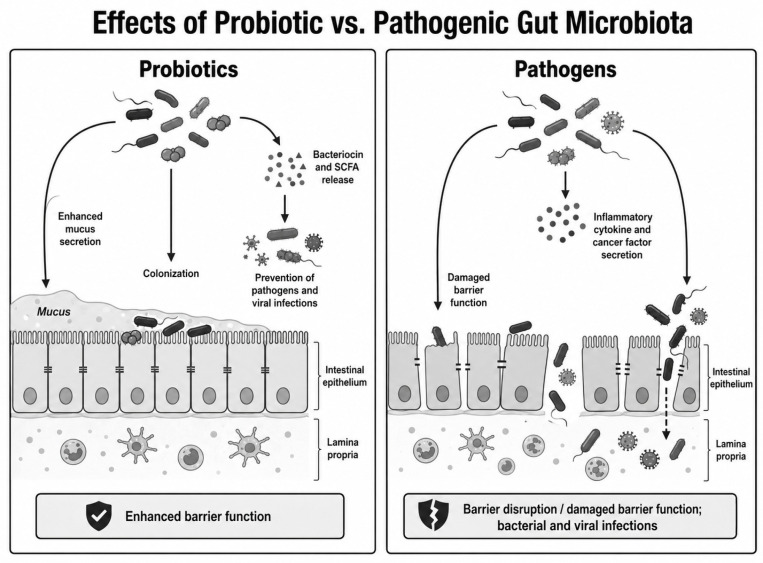
Schematic comparison of the effects of probiotic and pathogenic microorganisms on the intestinal epithelium. Probiotics may support intestinal barrier function by promoting colonization resistance, enhancing mucus secretion, strengthening epithelial integrity, and releasing antimicrobial substances and metabolites, including bacteriocins and short-chain fatty acids (SCFAs). These effects may reduce pathogen adhesion, bacterial and viral invasion, intestinal permeability, and inflammatory activation. In contrast, pathogenic gut microorganisms may impair epithelial barrier function, reduce barrier integrity, promote secretion of inflammatory cytokines and other harmful mediators, and facilitate bacterial or viral translocation into the lamina propria. These processes may contribute to intestinal inflammation, metabolic endotoxemia, chronic low-grade inflammation, and MetS-related dysfunction. SCFAs, short-chain fatty acids.

Specifically, probiotics containing *Bifidobacterium* have been associated with reduced inflammation, improved insulin sensitivity, enhanced lipid metabolism, and glycemic control [93] (Figure 5).

The study by Cani et al. showed that administration of the prebiotic oligofructose (OFS) to obese mice increased Bifidobacterium levels, reduced endotoxemia, and improved glucose tolerance in mice fed a high-fat diet [94].

Similarly, the study by Horvath et al. showed that consumption of trans-galacto-oligosaccharides (GOSs) improved insulin and lipid metabolism, increasing the abundance of Bifidobacterium and decreasing that of Gram-negative bacteria such as Bacteroides spp., Desulfovibrio spp., and the *C. histolyticum* group, without significant changes in peripheral insulin sensitivity. Alpha-galacto-oligosaccharides were associated with a dose-dependent reduction in appetite and food intake [84].

Both studies suggest that increased Bifidobacteria are associated with improvements in glucose and insulin metabolism. Interestingly, OFS was associated with a clear improvement in glucose tolerance, whereas trans-GOS improved lipid metabolism but did not exert the same effect on peripheral insulin sensitivity. This suggests that different types of prebiotics may have distinct metabolic effects. Overall, prebiotics appear to modify gut microbiota composition, but their effects on MetS and body weight remain under investigation.

To improve clarity, the main characteristics, mechanisms, and reported metabolic effects of probiotics, prebiotics, and synbiotics are summarized in Table 1.

Postbiotics are preparations of inactivated microorganisms and/or their components that confer a health benefit on the host [96]. They may include heat-killed bacteria, bacterial cell-wall components, enzymes, peptides, extracellular vesicles, organic acids, SCFAs, and other bioactive metabolites [96,97]. Compared with live probiotics, postbiotics may offer advantages regarding stability, standardization, storage, and safety, particularly in vulnerable populations [96,98]. In the context of MetS, postbiotics may influence host metabolism through modulation of intestinal barrier integrity, immune signaling, oxidative stress, bile acid metabolism, SCFA-related pathways, and low-grade inflammation [72,97]. However, clinical evidence remains less mature than for conventional probiotics and prebiotics, and further well-designed randomized controlled trials are required [72,96].

Next-generation probiotics are also emerging as a promising therapeutic approach [99,100]. Unlike traditional probiotic strains, these candidates are usually selected based on metagenomic, metabolomic, or mechanistic evidence linking them to host metabolic or immune regulation [99,101]. Examples include *Akkermansia muciniphila*, *Faecalibacterium prausnitzii*, and other commensal taxa associated with gut barrier function, anti-inflammatory activity, and metabolic homeostasis [102,103]. Nevertheless, their clinical use in obesity and MetS remains investigational, and important issues related to strain selection, formulation, safety, colonization, regulatory classification, and long-term efficacy remain unresolved [99,100,102].

### 7.3. Fecal Microbiota Transplantation (FMT)

Fecal microbiota transplantation (FMT) is a therapeutic procedure that involves transferring fecal material from a healthy donor to a recipient, with the aim of improving or restoring gut microbiota composition and, consequently, treating disease [104]. In recent years, researchers have explored its potential to improve MetS and, by extension, obesity [105]. Human studies of FMT in obesity and MetS have produced mixed results [106,107,108]. Some trials and systematic reviews have reported short-term improvements in selected glycemic, insulin-resistance, intestinal permeability, and lipid-related parameters, including fasting glucose, HbA1c, HDL cholesterol, and insulin sensitivity, particularly when lean donor microbiota are transferred to recipients with obesity or metabolic impairment [106,107,109,110,111]. However, effects on body weight are generally inconsistent, and the durability of metabolic benefits remains uncertain [106,108,112]. Response to FMT appears to depend on donor microbial composition, recipient baseline microbiota, engraftment efficiency, route of administration, bowel preparation, diet, and follow-up duration [45,107,110,112]. FMT facilitates the transfer of beneficial bacteria to the gut and has been associated with reduced oxidative stress and chronic low-grade inflammation [113]. Therefore, FMT should currently be regarded as an experimental microbiota-targeted intervention for metabolic diseases rather than an established treatment, and larger controlled trials with longer follow-up are needed before routine clinical application [106,107].

Capsule administration appears to be associated with a low risk of adverse effects; however, in patients with impaired intestinal barrier integrity, the risk of infection from transplanted bacteria remains a concern [105].

FMT can transfer the metabolic phenotype of the donor, and its effectiveness depends largely on the baseline microbial composition of the recipient’s gut microbiota [114].

Experimental studies support these findings. In particular, Ridaura et al. showed that transfer of microbiota from obese humans to germ-free mice led to increased fat mass compared with transfer of microbiota from lean humans. Furthermore, cohabitation of mice carrying microbiota from obese donors with mice that had received microbiota from lean humans reduced the emergence of the obese phenotype.

In parallel, Bäckhed et al. showed that colonizing germ-free mice with gut microbiota through fecal material from conventional mice resulted in increased adiposity and reduced insulin sensitivity, despite reduced food intake. Similarly, mice that received microbiota from obese ob/ob mice showed greater weight gain than those that received microbiota from lean mice [40,43,115].

Gut bacterial composition differs between lean and overweight hosts, and gut bacteria appear to contribute to weight gain and fat mass through metabolic, inflammatory, and neural pathways. When gut bacteria from mice that had undergone bariatric surgery were transferred by fecal transplantation to germ-free mice, the latter exhibited genes associated with a lean phenotype. Changes in microbial populations after bariatric surgery resulted in enhanced bile salt metabolism and improved glucose tolerance [116].

Finally, diet influences both metabolic disease and gut microbiota composition, and dietary modification represents a safe intervention for modulating the microbiota and improving metabolic control.

### 7.4. Personalized Nutrition and Microbiome-Informed Interventions

Personalized nutrition represents another emerging microbiome-targeted strategy [76,117]. Rather than applying uniform dietary advice, this approach uses individual characteristics such as gut microbiome composition, dietary habits, anthropometrics, glycemic responses, lipid responses, genetic background, and clinical phenotype to tailor dietary recommendations [117,118,119]. This may be particularly relevant for MetS, where individuals differ substantially in postprandial glucose and triglyceride responses, insulin resistance, visceral adiposity, and microbiota-derived metabolite profiles [76,118]. Microbiome-informed nutrition may help identify individuals more likely to respond to fiber-rich diets, prebiotics, polyphenols, or other dietary interventions [120,121]. However, although early trials are promising, implementation in routine clinical practice requires further validation, standardization of microbiome testing, cost-effectiveness assessment, and evidence that personalized approaches improve long-term MetS outcomes beyond standard dietary counseling [76,122]. The most important microbiome-targeted interventions are depicted in Table 2.

Overall, microbiome-targeted therapies for MetS are promising but remain heterogeneous in their clinical effects. Dietary fiber and prebiotic-rich diets have the strongest biological plausibility and safety profile, while probiotics and synbiotics show strain- and formulation-specific benefits. FMT, postbiotics, next-generation probiotics, and personalized nutrition are emerging approaches, but their routine clinical use requires larger randomized trials, standardized microbiome and metabolomic endpoints, long-term follow-up, and better identification of responder phenotypes.

## 8. Discussion

The gut microbiota plays an important role in glucose and lipid metabolism, as well as in immune system regulation [4]. Alterations in gut microbiota composition and function can significantly affect host metabolism and may contribute to the development of obesity, insulin resistance, and metabolic syndrome (MetS) [18,26]. Diet, particularly dietary fiber intake, is one of the major determinants of gut microbiota composition [21,123]. Bacterial fermentation of dietary fiber in the colon increases the production of short-chain fatty acids (SCFAs), which represent important mediators of host–microbiome interactions [23,124].

Taken together, the available evidence suggests that gut microbiota alterations in MetS should not be interpreted solely through individual taxonomic shifts, such as the Firmicutes/Bacteroidetes ratio [39,43,125]. Instead, they should be interpreted through integrated functional pathways involving microbial metabolites, intestinal permeability, inflammatory signaling, bile acid metabolism, gut–brain axis regulation, and host metabolic phenotype [4,19,26,126].

SCFAs act as regulators of intestinal epithelial cell function and differentiation, thereby contributing to the maintenance of intestinal barrier integrity [23,124]. However, some SCFA-related metabolites, particularly acetate, may also participate in hepatic lipogenesis under specific nutritional and metabolic conditions. Specifically, unabsorbed fructose can be metabolized to acetate by the intestinal microbiota, transported to the liver, and converted to acetyl-CoA by acyl-CoA synthetase short-chain family member 2 (ACSS2), thereby bypassing the classical ATP citrate lyase (ACLY) pathway [51]. The rate of fructose intake appears to be important, as gradual consumption favors absorption in the small intestine and may involve both classical and alternative pathways in lipogenesis [51].

In a related study, butyrate was shown to affect lipid metabolism in intestinal Caco-2 cells by significantly reducing the synthesis and secretion of chylomicrons and very low-density lipoproteins (VLDLs). At the same time, butyrate inhibited the synthesis of apolipoprotein B-48, contributing to the regulation of intestinal fat absorption and circulating lipoprotein concentrations [127].

ACSS2 is a key enzyme in the conversion of acetate to lipogenic acetyl-CoA, highlighting its role in microbiota-associated acetate metabolism and host lipogenesis [51]. However, ACSS2 appears to have a dual role in energy metabolism. During periods of energy surplus, it may promote fat storage in adipose tissue, whereas during fasting, it may contribute to the mobilization and utilization of stored fat. In experimental models, the absence of ACSS2 was associated with reduced body weight and attenuation of diet-induced hepatic steatosis. Furthermore, ACSS2 deficiency reduced dietary lipid absorption from the intestine and disrupted the redistribution and utilization of triglycerides between adipose tissue and liver by lowering the expression of genes involved in fatty acid transport and oxidation [83,128]. Overall, these findings suggest that ACSS2 plays an important role in host metabolic pathways and may represent a potential therapeutic target for metabolic disorders.

Recent advances in multi-omics technologies have also changed the interpretation of microbiome research. Metagenomics, metatranscriptomics, metabolomics, proteomics, and integrated systems biology approaches allow a more functional assessment of the microbiome than taxonomic profiling alone [126]. This is particularly relevant in MetS, where microbial taxa may vary between populations, whereas functional pathways such as SCFA production, bile acid transformation, trimethylamine/trimethylamine N-oxide (TMA/TMAO) metabolism, lipopolysaccharide (LPS)-related inflammatory signaling, and host–microbiome metabolic interactions may be more reproducible and clinically informative [126,129]. However, standardization of sampling, sequencing, bioinformatic analysis, and validation across independent cohorts remains essential before multi-omics signatures can be translated into clinical biomarkers or personalized interventions [126,130].

This review has several limitations. First, it was designed as a narrative review rather than a systematic review or meta-analysis. Although a structured literature search was performed, the selection and interpretation of studies may be influenced by selection bias, and no formal risk-of-bias assessment or quantitative synthesis was conducted. Therefore, the conclusions should be interpreted as a critical synthesis of the available literature rather than as a definitive estimate of effect size or causality [5,126].

Second, the available evidence is heterogeneous with respect to study design, population characteristics, microbiome assessment methods, sequencing platforms, bioinformatic pipelines, dietary background, medication exposure, and clinical definitions of MetS. These differences limit direct comparison between studies and may partly explain inconsistent findings, such as those reported for the *Firmicutes*/*Bacteroidetes* ratio [126,130].

Third, many human studies have been conducted in Western or high-income populations, where dietary patterns, lifestyle, obesity prevalence, medication use, and environmental exposures may differ substantially from those in other regions. Therefore, the generalizability of some findings to non-Western populations, different ethnic groups, and populations with different dietary traditions remains limited [131].

Finally, it is difficult to isolate the independent contribution of the gut microbiota from other factors that influence MetS. Diet, age, sex, genetic background, adiposity, physical activity, medication use, comorbidities, and host inflammatory status can all shape both gut microbiota composition and metabolic phenotype [132,133,134]. As a result, many reported associations may be bidirectional or confounded, and causality remains difficult to establish in human observational studies. Future research should prioritize large, longitudinal, multi-ethnic cohorts, standardized microbiome and metabolomic methods, mechanistic studies, and well-designed randomized controlled trials to clarify causal pathways and identify clinically relevant microbiome-based interventions [126,130].

## 9. Conclusions

Overall, the available evidence highlights the central role of the gut microbiota in the regulation of lipid and glucose metabolism, intestinal barrier integrity, immune activation, bile acid signaling, and gut–brain axis communication. Through these mechanisms, gut microbiota dysbiosis may contribute not only to obesity and type 2 diabetes mellitus, but also to the broader pathophysiology of MetS. Altered SCFA production, increased intestinal permeability, metabolic endotoxemia, chronic low-grade inflammation, changes in bile acid metabolism, and microbiota-related acetate/ACSS2-dependent lipogenesis may promote insulin resistance, central adiposity, dyslipidemia, hepatic steatosis, impaired glucose homeostasis, hypertension, and increased cardiometabolic risk.

Therefore, MetS should be considered an integrated clinical outcome of multiple microbiota-related metabolic, inflammatory, and neuroendocrine pathways rather than a collection of isolated abnormalities. Targeted nutritional and therapeutic interventions, including diets rich in plant fibers, prebiotics, probiotics, synbiotics, and other microbiota-modulating strategies, may represent promising approaches for improving gut microbiota composition and function. These interventions may help prevent or ameliorate several MetS components, although further well-designed human studies are required to clarify causality, identify responders, and define the optimal intervention type, dose, and duration.

## Figures and Tables

**Table 1:** Summary of probiotics, prebiotics, and synbiotics in MetS and obesity.

Intervention	Examples	Main Proposed Mechanisms	Reported Metabolic Effects	Key Supporting References
Probiotics	*Lactobacillus* spp., *Bifidobacterium* spp., *Lactobacillus gasseri*	Modulation of gut microbiota composition; strengthening of the intestinal barrier; increased SCFA production; reduced endotoxemia; stimulation of anorexigenic hormones such as GLP-1 and PYY	Potential reduction in body weight and abdominal adiposity; improved glucose metabolism, insulin resistance, HbA1c, and lipid profile	[5,66,74,86,87,88,92,93]
Prebiotics	Inulin, oligofructose, fructo-oligosaccharides, galacto-oligosaccharides, resistant starch	Selective stimulation of beneficial bacteria, particularly *Bifidobacterium* and *Lactobacillus*; increased SCFA production; reduced intestinal permeability and endotoxemia	Improved glucose tolerance and lipid metabolism in some studies; possible appetite reduction; effects on body weight remain under investigation	[46,84,85,95]
Synbiotics	Combination of probiotics and prebiotics	Synergistic enhancement of probiotic survival and activity; combined modulation of microbial composition and metabolite production	Potential improvement in inflammatory, glycemic, and lipid parameters, although effects may depend on strain, dose, duration, and host characteristics	[20,53,54,74,92]
Main limitations	—	Heterogeneity of strains, doses, intervention duration, and study populations; stronger evidence from animal studies than human trials	Clinical efficacy remains variable; further well-designed randomized controlled trials are required	[74,88,92]

**Table 2:** Representative clinical evidence for microbiome-targeted interventions in obesity and MetS.

Intervention Type	Representative Examples	Population/ Study Type	Main Reported Outcomes	Evidence Strength	Key Supporting References
**Dietary fiber/prebiotic-rich diets**	Plant fiber, inulin, oligofructose, fructo-oligosaccharides, galacto-oligosaccharides, resistant starch	Individuals with obesity, insulin resistance, T2DM, or MetS-related risk factors; randomized and dietary intervention studies	Increased SCFA production, enrichment of beneficial taxa such as *Bifidobacterium*, improved gut barrier function, possible improvements in glucose tolerance, appetite regulation, and lipid metabolism	Moderate; biologically plausible and supported by several studies, but responses vary by fiber type, baseline diet, and microbiota	[23,95,123]
**Traditional probiotics**	*Lactobacillus* spp., *Bifidobacterium* spp., *Lactobacillus casei*, *Lactobacillus gasseri*, mixed strains	Adults with obesity, MetS, T2DM, or cardiometabolic risk; randomized trials and meta-analyses	Potential reductions in BMI, waist circumference, fasting glucose, HOMA-IR, inflammatory markers, and selected lipid parameters	Moderate but heterogeneous; effects are strain-, dose-, and duration-dependent	[74,88,92]
**Synbiotics**	Probiotic strains combined with prebiotics such as FOS or inulin	Patients with obesity, T2DM, MAFLD, or MetS-related abnormalities; randomized trials and meta-analyses	Potential improvements in glycemic control, lipid profile, inflammatory markers, and gut microbiota composition	Low-to-moderate to moderate; promising, but formulations and populations differ considerably	[74,92]
**Postbiotics**	Heat-killed bacteria, microbial metabolites, SCFAs, bacterial components	Emerging clinical and experimental evidence in obesity and metabolic disorders	May improve intestinal barrier integrity, inflammation, oxidative stress, and metabolic signaling without requiring live bacterial administration	Low-to-moderate; promising but fewer large trials in MetS	[72,96,97,98]
**Next-generation probiotics**	*Akkermansia muciniphila*, *Faecalibacterium prausnitzii*, other metabolically relevant commensals	Early-phase human studies and mechanistic studies; mostly investigational	Potential improvement in gut barrier function, inflammation, insulin sensitivity, and metabolic regulation	Emerging; requires further safety and efficacy validation	[99,100,102,103]
**FMT**	Lean-donor FMT; oral capsules or endoscopic administration	Patients with obesity, insulin resistance, or MetS; small randomized trials and systematic reviews	Some improvements in insulin sensitivity, glucose metabolism, intestinal permeability, or lipid-related outcomes; inconsistent effects on body weight	Low-to-moderate; experimental, limited by small trials and variable engraftment	[29,106,109,110,112]
**Personalized nutrition based on microbiome/metabolic profiling**	Algorithms using microbiome, postprandial glucose/lipid responses, diet, and clinical data	Adults with cardiometabolic risk; randomized and observational precision-nutrition studies	Improved individualized dietary matching; potential improvements in postprandial glycemia, triglyceride responses, and cardiometabolic risk factors	Emerging to moderate; promising but requires long-term MetS outcome validation	[76,117,118,120,122]

## Data Availability

No new data were created.

## References

[1] Ahmed S.K., Mohammed R.A. (2025). Obesity: Prevalence, causes, consequences, management, preventive strategies and future research directions. Metabol. Open.

[2] Durma A.C. (2024). The Role of Gut Microbiota in Obesity. Adv. Microbiol..

[3] Yarahmadi A., Afkhami H., Javadi A., Kashfi M. (2024). Understanding the complex function of gut microbiota: Its impact on the pathogenesis of obesity and beyond: A comprehensive review. Diabetol. Metab. Syndr..

[4] de Vos W.M., Tilg H., Van Hul M., Cani P.D. (2022). Gut microbiome and health: Mechanistic insights. Gut.

[5] Green M., Arora K., Prakash S. (2020). Microbial Medicine: Prebiotic and Probiotic Functional Foods to Target Obesity and Metabolic Syndrome. Int. J. Mol. Sci..

[6] Xu Z., Jiang W., Huang W., Lin Y., Chan F.K.L., Ng S.C. (2022). Gut microbiota in patients with obesity and metabolic disorders—A systematic review. Genes Nutr..

[7] Hernandez M.A.G., Canfora E.E., Jocken J.W.E., Blaak E.E. (2019). The Short-Chain Fatty Acid Acetate in Body Weight Control and Insulin Sensitivity. Nutrients.

[8] Agusti A., Garcia-Pardo M.P., Lopez-Almela I., Campillo I., Maes M., Romani-Perez M., Sanz Y. (2018). Interplay Between the Gut-Brain Axis, Obesity and Cognitive Function. Front. Neurosci..

[9] Herath H.M.M., Weerasinghe N.P., Weerarathna T.P., Amarathunga A. (2018). A Comparison of the Prevalence of the Metabolic Syndrome among Sri Lankan Patients with Type 2 Diabetes Mellitus Using WHO, NCEP-ATP III, and IDF Definitions. Int. J. Chronic Dis..

[10] Alberti K.G., Zimmet P.Z. (1998). Definition, diagnosis and classification of diabetes mellitus and its complications. Part 1: Diagnosis and classification of diabetes mellitus provisional report of a WHO consultation. Diabet. Med..

[11] Balkau B., Charles M.A. (1999). Comment on the provisional report from the WHO consultation. European Group for the Study of Insulin Resistance (EGIR). Diabet. Med..

[12] Alberti K.G., Zimmet P., Shaw J. (2006). Metabolic syndrome--a new world-wide definition. A Consensus Statement from the International Diabetes Federation. Diabet. Med..

[13] Expert Panel on Detection, Evaluation, and Treatment of High Blood Cholesterol in Adults (2001). Executive Summary of The Third Report of The National Cholesterol Education Program (NCEP) Expert Panel on Detection, Evaluation, and Treatment of High Blood Cholesterol in Adults (Adult Treatment Panel III). JAMA.

[14] Kassi E., Pervanidou P., Kaltsas G., Chrousos G. (2011). Metabolic syndrome: Definitions and controversies. BMC Med..

[15] Huang P.L. (2009). A comprehensive definition for metabolic syndrome. Dis. Model. Mech..

[16] Grundy S.M., Cleeman J.I., Daniels S.R., Donato K.A., Eckel R.H., Franklin B.A., Gordon D.J., Krauss R.M., Savage P.J., Smith S.C. (2005). Diagnosis and management of the metabolic syndrome: An American Heart Association/National Heart, Lung, and Blood Institute Scientific Statement. Circulation.

[17] Chackrewarthy S., Gunasekera D., Pathmeswaren A., Wijekoon C.N., Ranawaka U.K., Kato N., Takeuchi F., Wickremasinghe A.R. (2013). A Comparison between Revised NCEP ATP III and IDF Definitions in Diagnosing Metabolic Syndrome in an Urban Sri Lankan Population: The Ragama Health Study. ISRN Endocrinol..

[18] Cani P.D., Amar J., Iglesias M.A., Poggi M., Knauf C., Bastelica D., Neyrinck A.M., Fava F., Tuohy K.M., Chabo C. (2007). Metabolic endotoxemia initiates obesity and insulin resistance. Diabetes.

[19] Mohammad S., Thiemermann C. (2020). Role of Metabolic Endotoxemia in Systemic Inflammation and Potential Interventions. Front. Immunol..

[20] McBurney M.I., Cho C.E. (2024). Understanding the role of the human gut microbiome in overweight and obesity. Ann. N. Y. Acad. Sci..

[21] Flint H.J., Scott K.P., Duncan S.H., Louis P., Forano E. (2012). Microbial degradation of complex carbohydrates in the gut. Gut Microbes.

[22] Chen Z., Liang N., Zhang H., Li H., Guo J., Zhang Y., Chen Y., Wang Y., Shi N. (2024). Resistant starch and the gut microbiome: Exploring beneficial interactions and dietary impacts. Food Chem. X.

[23] den Besten G., van Eunen K., Groen A.K., Venema K., Reijngoud D.J., Bakker B.M. (2013). The role of short-chain fatty acids in the interplay between diet, gut microbiota, and host energy metabolism. J. Lipid Res..

[24] Cheng Z., Zhang L., Yang L., Chu H. (2022). The critical role of gut microbiota in obesity. Front. Endocrinol..

[25] Suganami T., Tanimoto-Koyama K., Nishida J., Itoh M., Yuan X., Mizuarai S., Kotani H., Yamaoka S., Miyake K., Aoe S. (2007). Role of the Toll-like receptor 4/NF-kappaB pathway in saturated fatty acid-induced inflammatory changes in the interaction between adipocytes and macrophages. Arterioscler. Thromb. Vasc. Biol..

[26] Cani P.D., Bibiloni R., Knauf C., Waget A., Neyrinck A.M., Delzenne N.M., Burcelin R. (2008). Changes in gut microbiota control metabolic endotoxemia-induced inflammation in high-fat diet-induced obesity and diabetes in mice. Diabetes.

[27] Phuong-Nguyen K., McNeill B.A., Aston-Mourney K., Rivera L.R. (2023). Advanced Glycation End-Products and Their Effects on Gut Health. Nutrients.

[28] Snelson M., Coughlan M.T. (2019). Dietary Advanced Glycation End Products: Digestion, Metabolism and Modulation of Gut Microbial Ecology. Nutrients.

[29] Qu W., Yuan X., Zhao J., Zhang Y., Hu J., Wang J., Li J. (2017). Dietary advanced glycation end products modify gut microbial composition and partially increase colon permeability in rats. Mol. Nutr. Food Res..

[30] Jansen F.A.C., Fogliano V., Rubert J., Hoppenbrouwers T. (2023). Dietary Advanced Glycation End. products interacting with the intestinal epithelium: What do we really know?. Mol. Metab..

[31] Dong H., Zhang Y., Huang Y., Deng H. (2022). Pathophysiology of RAGE in inflammatory diseases. Front. Immunol..

[32] Wang J., Cai W., Yu J., Liu H., He S., Zhu L., Xu J. (2022). Dietary Advanced Glycation End Products Shift the Gut Microbiota Composition and Induce Insulin Resistance in Mice. Diabetes Metab. Syndr. Obes..

[33] Aschner M., Skalny A.V., Gritsenko V.A., Kartashova O.L., Santamaria A., Rocha J.B.T., Spandidos D.A., Zaitseva I.P., Tsatsakis A., Tinkov A.A. (2023). Role of gut microbiota in the modulation of the health effects of advanced glycation end-products (Review). Int. J. Mol. Med..

[34] Machate D.J., Figueiredo P.S., Marcelino G., Guimaraes R.C.A., Hiane P.A., Bogo D., Pinheiro V.A.Z., Oliveira L.C.S., Pott A. (2020). Fatty Acid Diets: Regulation of Gut Microbiota Composition and Obesity and Its Related Metabolic Dysbiosis. Int. J. Mol. Sci..

[35] Leonard M.M., Valitutti F., Karathia H., Pujolassos M., Kenyon V., Fanelli B., Troisi J., Subramanian P., Camhi S., Colucci A. (2021). Microbiome signatures of progression toward celiac disease onset in at-risk children in a longitudinal prospective cohort study. Proc. Natl. Acad. Sci. USA.

[36] Tilg H., Adolph T.E., Gerner R.R., Moschen A.R. (2018). The Intestinal Microbiota in Colorectal Cancer. Cancer Cell.

[37] Loomba R., Seguritan V., Li W., Long T., Klitgord N., Bhatt A., Dulai P.S., Caussy C., Bettencourt R., Highlander S.K. (2017). Gut Microbiome-Based Metagenomic Signature for Non-invasive Detection of Advanced Fibrosis in Human Nonalcoholic Fatty Liver Disease. Cell Metab..

[38] Qin J., Li R., Raes J., Arumugam M., Burgdorf K.S., Manichanh C., Nielsen T., Pons N., Levenez F., Yamada T. (2010). A human gut microbial gene catalogue established by metagenomic sequencing. Nature.

[39] Ley R.E., Backhed F., Turnbaugh P., Lozupone C.A., Knight R.D., Gordon J.I. (2005). Obesity alters gut microbial ecology. Proc. Natl. Acad. Sci. USA.

[40] Ridaura V.K., Faith J.J., Rey F.E., Cheng J., Duncan A.E., Kau A.L., Griffin N.W., Lombard V., Henrissat B., Bain J.R. (2013). Gut microbiota from twins discordant for obesity modulate metabolism in mice. Science.

[41] Fei N., Zhao L. (2013). An opportunistic pathogen isolated from the gut of an obese human causes obesity in germfree mice. ISME J..

[42] Andoh A., Nishida A., Takahashi K., Inatomi O., Imaeda H., Bamba S., Kito K., Sugimoto M., Kobayashi T. (2016). Comparison of the gut microbial community between obese and lean peoples using 16S gene sequencing in a Japanese population. J. Clin. Biochem. Nutr..

[43] Turnbaugh P.J., Ley R.E., Mahowald M.A., Magrini V., Mardis E.R., Gordon J.I. (2006). An obesity-associated gut microbiome with increased capacity for energy harvest. Nature.

[44] Li X.M., Lv Q., Chen Y.J., Yan L.B., Xiong X. (2024). Association between childhood obesity and gut microbiota: 16S rRNA gene sequencing-based cohort study. World J. Gastroenterol..

[45] Zhang S., Dang Y. (2022). Roles of gut microbiota and metabolites in overweight and obesity of children. Front. Endocrinol..

[46] Soldavini J., Kaunitz J.D. (2013). Pathobiology and potential therapeutic value of intestinal short-chain fatty acids in gut inflammation and obesity. Dig. Dis. Sci..

[47] Portincasa P., Bonfrate L., Vacca M., De Angelis M., Farella I., Lanza E., Khalil M., Wang D.Q., Sperandio M., Di Ciaula A. (2022). Gut Microbiota and Short Chain Fatty Acids: Implications in Glucose Homeostasis. Int. J. Mol. Sci..

[48] Morrison D.J., Preston T. (2016). Formation of short chain fatty acids by the gut microbiota and their impact on human metabolism. Gut Microbes.

[49] de La Serre C.B., Ellis C.L., Lee J., Hartman A.L., Rutledge J.C., Raybould H.E. (2010). Propensity to high-fat diet-induced obesity in rats is associated with changes in the gut microbiota and gut inflammation. Am. J. Physiol. Gastrointest. Liver Physiol..

[50] Barrea L., Muscogiuri G., Annunziata G., Laudisio D., Pugliese G., Salzano C., Colao A., Savastano S. (2019). From gut microbiota dysfunction to obesity: Could short-chain fatty acids stop this dangerous course?. Hormones.

[51] Zhao S., Jang C., Liu J., Uehara K., Gilbert M., Izzo L., Zeng X., Trefely S., Fernandez S., Carrer A. (2020). Dietary fructose feeds hepatic lipogenesis via microbiota-derived acetate. Nature.

[52] Wu H., Tremaroli V., Schmidt C., Lundqvist A., Olsson L.M., Kramer M., Gummesson A., Perkins R., Bergstrom G., Backhed F. (2020). The Gut Microbiota in Prediabetes and Diabetes: A Population-Based Cross-Sectional Study. Cell Metab..

[53] Forslund K., Hildebrand F., Nielsen T., Falony G., Le Chatelier E., Sunagawa S., Prifti E., Vieira-Silva S., Gudmundsdottir V., Pedersen H.K. (2015). Disentangling type 2 diabetes and metformin treatment signatures in the human gut microbiota. Nature.

[54] Bryrup T., Thomsen C.W., Kern T., Allin K.H., Brandslund I., Jorgensen N.R., Vestergaard H., Hansen T., Hansen T.H., Pedersen O. (2019). Metformin-induced changes of the gut microbiota in healthy young men: Results of a non-blinded, one-armed intervention study. Diabetologia.

[55] Wu H., Esteve E., Tremaroli V., Khan M.T., Caesar R., Manneras-Holm L., Stahlman M., Olsson L.M., Serino M., Planas-Felix M. (2017). Metformin alters the gut microbiome of individuals with treatment-naive type 2 diabetes, contributing to the therapeutic effects of the drug. Nat. Med..

[56] Mueller N.T., Differding M.K., Zhang M., Maruthur N.M., Juraschek S.P., Miller E.R., Appel L.J., Yeh H.C. (2021). Metformin Affects Gut Microbiome Composition and Function and Circulating Short-Chain Fatty Acids: A Randomized Trial. Diabetes Care.

[57] Afsar B., Afsar R.E., Lentine K.L. (2024). The impact of sodium-glucose cotransporter inhibitors on gut microbiota: A scoping review. J. Diabetes Metab. Disord..

[58] Kanbay M., Al-Shiab R., Shah E., Ozbek L., Guldan M., Ortiz A., Fouque D. (2025). Gut microbiota modulation in GLP-1RA and SGLT-2i therapy: Clinical implications and mechanistic insights in type 2 diabetes. Clin. Kidney J..

[59] Deng L., Yang Y., Xu G. (2022). Empagliflozin ameliorates type 2 diabetes mellitus-related diabetic nephropathy via altering the gut microbiota. Biochim. Biophys. Acta Mol. Cell Biol. Lipids.

[60] Mindrescu N.M., Guja C., Jinga V., Ispas S., Curici A., Nelson Twakor A., Pantea Stoian A.M. (2024). Interactions between Gut Microbiota and Oral Antihyperglycemic Drugs: A Systematic Review. Int. J. Mol. Sci..

[61] Gofron K.K., Wasilewski A., Malgorzewicz S. (2025). Effects of GLP-1 Analogues and Agonists on the Gut Microbiota: A Systematic Review. Nutrients.

[62] Guney-Coskun M., Basaranoglu M. (2024). Interplay of gut microbiota, glucagon-like peptide receptor agonists, and nutrition: New frontiers in metabolic dysfunction-associated steatotic liver disease therapy. World J. Gastroenterol..

[63] Moreira G.V., Azevedo F.F., Ribeiro L.M., Santos A., Guadagnini D., Gama P., Liberti E.A., Saad M., Carvalho C. (2018). Liraglutide modulates gut microbiota and reduces NAFLD in obese mice. J. Nutr. Biochem..

[64] Zhao L., Qiu Y., Zhang P., Wu X., Zhao Z., Deng X., Yang L., Wang D., Yuan G. (2022). Gut microbiota mediates positive effects of liraglutide on dyslipidemia in mice fed a high-fat diet. Front. Nutr..

[65] Shapiro H., Kolodziejczyk A.A., Halstuch D., Elinav E. (2018). Bile acids in glucose metabolism in health and disease. J. Exp. Med..

[66] Sun L., Xie C., Wang G., Wu Y., Wu Q., Wang X., Liu J., Deng Y., Xia J., Chen B. (2018). Gut microbiota and intestinal FXR mediate the clinical benefits of metformin. Nat. Med..

[67] Pimentel G.D., Micheletti T.O., Pace F., Rosa J.C., Santos R.V., Lira F.S. (2012). Gut-central nervous system axis is a target for nutritional therapies. Nutr. J..

[68] Borgmann D., Ciglieri E., Biglari N., Brandt C., Cremer A.L., Backes H., Tittgemeyer M., Wunderlich F.T., Bruning J.C., Fenselau H. (2021). Gut-brain communication by distinct sensory neurons differently controls feeding and glucose metabolism. Cell Metab..

[69] Bai L., Mesgarzadeh S., Ramesh K.S., Huey E.L., Liu Y., Gray L.A., Aitken T.J., Chen Y., Beutler L.R., Ahn J.S. (2019). Genetic Identification of Vagal Sensory Neurons That Control Feeding. Cell.

[70] Berthoud H.R. (2008). The vagus nerve, food intake and obesity. Regul. Pept..

[71] Dockray G.J. (2013). Enteroendocrine cell signalling via the vagus nerve. Curr. Opin. Pharmacol..

[72] Li H.Y., Zhou D.D., Gan R.Y., Huang S.Y., Zhao C.N., Shang A., Xu X.Y., Li H.B. (2021). Effects and Mechanisms of Probiotics, Prebiotics, Synbiotics, and Postbiotics on Metabolic Diseases Targeting Gut Microbiota: A Narrative Review. Nutrients.

[73] Ahmed N., Gaur V., Kamle M., Chauhan A., Chauhan R., Kumar P., Singh N.A. (2025). Microbiome-based therapeutics for metabolic disorders: Harnessing microbial intrusions for treatment. Front. Med. Technol..

[74] Chen T., Wang J., Liu Z., Gao F. (2023). Effect of supplementation with probiotics or synbiotics on cardiovascular risk factors in patients with metabolic syndrome: A systematic review and meta-analysis of randomized clinical trials. Front. Endocrinol..

[75] Al-Juhani A., Desoky M.S., Almuhaimid A.A., Zaheer M., Alhaqbani H.F., Abalkhail E.A., Alanazi S.A., Alzahrani R.S., Alrefaai M., Desoky R. (2025). Efficacy of Gut Microbiome-Targeted Therapies in Modulating Systemic Inflammation and Low-Grade Chronic Inflammatory States in Adults with Metabolic Disorders: A Systematic Review. Cureus.

[76] Plaza-Diaz J., Herrera-Quintana L., Olivares-Arancibia J., Vazquez-Lorente H. (2026). Personalized Nutrition Through the Gut Microbiome in Metabolic Syndrome and Related Comorbidities. Nutrients.

[77] Makki K., Deehan E.C., Walter J., Backhed F. (2018). The Impact of Dietary Fiber on Gut Microbiota in Host Health and Disease. Cell Host Microbe.

[78] Cronin P., Joyce S.A., O’Toole P.W., O’Connor E.M. (2021). Dietary Fibre Modulates the Gut Microbiota. Nutrients.

[79] Garcia-Mantrana I., Selma-Royo M., Alcantara C., Collado M.C. (2018). Shifts on Gut Microbiota Associated to Mediterranean Diet Adherence and Specific Dietary Intakes on General Adult Population. Front. Microbiol..

[80] David L.A., Maurice C.F., Carmody R.N., Gootenberg D.B., Button J.E., Wolfe B.E., Ling A.V., Devlin A.S., Varma Y., Fischbach M.A. (2014). Diet rapidly and reproducibly alters the human gut microbiome. Nature.

[81] De Filippo C., Cavalieri D., Di Paola M., Ramazzotti M., Poullet J.B., Massart S., Collini S., Pieraccini G., Lionetti P. (2010). Impact of diet in shaping gut microbiota revealed by a comparative study in children from Europe and rural Africa. Proc. Natl. Acad. Sci. USA.

[82] Igudesman D., Crandell J.L., Corbin K.D., Hooper J., Thomas J.M., Bulik C.M., Pence B.W., Pratley R.E., Kosorok M.R., Maahs D.M. (2023). Associations of Dietary Intake with the Intestinal Microbiota and Short-Chain Fatty Acids Among Young Adults with Type 1 Diabetes and Overweight or Obesity. J. Nutr..

[83] Huang Z., Zhang M., Plec A.A., Estill S.J., Cai L., Repa J.J., McKnight S.L., Tu B.P. (2018). ACSS2 promotes systemic fat storage and utilization through selective regulation of genes involved in lipid metabolism. Proc. Natl. Acad. Sci. USA.

[84] Horvath A., Zukauskaite K., Hazia O., Balazs I., Stadlbauer V. (2024). Human. gut microbiome: Therapeutic opportunities for metabolic syndrome-Hype or hope?. Endocrinol. Diabetes Metab..

[85] Roberfroid M., Gibson G.R., Hoyles L., McCartney A.L., Rastall R., Rowland I., Wolvers D., Watzl B., Szajewska H., Stahl B. (2010). Prebiotic effects: Metabolic and health benefits. Br. J. Nutr..

[86] Akbari V., Hendijani F. (2016). Effects of probiotic supplementation in patients with type 2 diabetes: Systematic review and meta-analysis. Nutr. Rev..

[87] Samah S., Ramasamy K., Lim S.M., Neoh C.F. (2016). Probiotics for the management of type 2 diabetes mellitus: A systematic review and meta-analysis. Diabetes Res. Clin. Pract..

[88] Koutnikova H., Genser B., Monteiro-Sepulveda M., Faurie J.M., Rizkalla S., Schrezenmeir J., Clement K. (2019). Impact of bacterial probiotics on obesity, diabetes and non-alcoholic fatty liver disease related variables: A systematic review and meta-analysis of randomised controlled trials. BMJ Open.

[89] Mederle A.L., Dima M., Stoicescu E.R., Capastraru B.F., Levai C.M., Hategan O.A., Maghiari A.L. (2024). Impact of Gut Microbiome Interventions on Glucose and Lipid Metabolism in Metabolic Diseases: A Systematic Review and Meta-Analysis. Life.

[90] Hadi A., Arab A., Khalesi S., Rafie N., Kafeshani M., Kazemi M. (2021). Effects of probiotic supplementation on anthropometric and metabolic characteristics in adults with metabolic syndrome: A systematic review and meta-analysis of randomized clinical trials. Clin. Nutr..

[91] Cao N., Zhao F., Kwok L.Y., Wang H., Sun Z. (2024). Impact of probiotics on weight loss, glucose and lipid metabolism in overweight or obese women: A meta-analysis of randomized controlled trials. Curr. Res. Food Sci..

[92] Tenorio-Jimenez C., Martinez-Ramirez M.J., Gil A., Gomez-Llorente C. (2020). Effects of Probiotics on Metabolic Syndrome: A Systematic Review of Randomized Clinical Trials. Nutrients.

[93] Gerard P. (2016). Gut microbiota and obesity. Cell. Mol. Life Sci..

[94] Cani P.D., Neyrinck A.M., Fava F., Knauf C., Burcelin R.G., Tuohy K.M., Gibson G.R., Delzenne N.M. (2007). Selective increases of bifidobacteria in gut microflora improve high-fat-diet-induced diabetes in mice through a mechanism associated with endotoxaemia. Diabetologia.

[95] Davani-Davari D., Negahdaripour M., Karimzadeh I., Seifan M., Mohkam M., Masoumi S.J., Berenjian A., Ghasemi Y. (2019). Prebiotics: Definition, Types, Sources, Mechanisms, and Clinical Applications. Foods.

[96] Salminen S., Collado M.C., Endo A., Hill C., Lebeer S., Quigley E.M.M., Sanders M.E., Shamir R., Swann J.R., Szajewska H. (2021). The International Scientific Association of Probiotics and Prebiotics (ISAPP) consensus statement on the definition and scope of postbiotics. Nat. Rev. Gastroenterol. Hepatol..

[97] Ragavan M.L., Hemalatha S. (2024). The functional roles of short chain fatty acids as postbiotics in human gut: Future perspectives. Food Sci. Biotechnol..

[98] Vinderola G., Benkowski A., Bernardeau M., Chenoll E., Collado M.C., Cronin U., Eckhardt E., Green J.B., Ipharraguerre I.R., Kemperman R. (2025). Postbiotics: A perspective on their quantification. Front. Nutr..

[99] Tiwari A., Ika Krisnawati D., Susilowati E., Mutalik C., Kuo T.R. (2024). Next-Generation Probiotics and Chronic Diseases: A Review of Current Research and Future Directions. J. Agric. Food Chem..

[100] Vallianou N.G., Kounatidis D., Tsilingiris D., Panagopoulos F., Christodoulatos G.S., Evangelopoulos A., Karampela I., Dalamaga M. (2023). The Role of Next-Generation Probiotics in Obesity and Obesity-Associated Disorders: Current Knowledge and Future Perspectives. Int. J. Mol. Sci..

[101] Cheng H.L., Yen G.C., Huang S.C., Chen S.C., Hsu C.L. (2022). The next generation beneficial actions of novel probiotics as potential therapeutic targets and prediction tool for metabolic diseases. J. Food Drug Anal..

[102] Depommier C., Everard A., Druart C., Plovier H., Van Hul M., Vieira-Silva S., Falony G., Raes J., Maiter D., Delzenne N.M. (2019). Supplementation with Akkermansia muciniphila in overweight and obese human volunteers: A proof-of-concept exploratory study. Nat. Med..

[103] Martin R., Rios-Covian D., Huillet E., Auger S., Khazaal S., Bermudez-Humaran L.G., Sokol H., Chatel J.M., Langella P. (2023). Faecalibacterium: A bacterial genus with promising human health applications. FEMS Microbiol. Rev..

[104] Wang J.W., Kuo C.H., Kuo F.C., Wang Y.K., Hsu W.H., Yu F.J., Hu H.M., Hsu P.I., Wang J.Y., Wu D.C. (2019). Fecal microbiota transplantation: Review and update. J. Formos. Med. Assoc..

[105] Chen C., Chen L., Sun D., Li C., Xi S., Ding S., Luo R., Geng Y., Bai Y. (2022). Adverse events of intestinal microbiota transplantation in randomized controlled trials: A systematic review and meta-analysis. Gut Pathog..

[106] Proenca I.M., Allegretti J.R., Bernardo W.M., de Moura D.T.H., Ponte Neto A.M., Matsubayashi C.O., Flor M.M., Kotinda A., de Moura E.G.H. (2020). Fecal microbiota transplantation improves metabolic syndrome parameters: Systematic review with meta-analysis based on randomized clinical trials. Nutr. Res..

[107] Qiu B., Liang J., Li C. (2023). Effects of fecal microbiota transplantation in metabolic syndrome: A meta-analysis of randomized controlled trials. PLoS ONE.

[108] Lahtinen P., Juuti A., Luostarinen M., Niskanen L., Liukkonen T., Tillonen J., Kossi J., Ilvesmaki V., Viljakka M., Satokari R. (2022). Effectiveness of Fecal Microbiota Transplantation for Weight Loss in Patients with Obesity Undergoing Bariatric Surgery: A Randomized Clinical Trial. JAMA Netw. Open.

[109] Vrieze A., Van Nood E., Holleman F., Salojarvi J., Kootte R.S., Bartelsman J.F., Dallinga-Thie G.M., Ackermans M.T., Serlie M.J., Oozeer R. (2012). Transfer of intestinal microbiota from lean donors increases insulin sensitivity in individuals with metabolic syndrome. Gastroenterology.

[110] Kootte R.S., Levin E., Salojarvi J., Smits L.P., Hartstra A.V., Udayappan S.D., Hermes G., Bouter K.E., Koopen A.M., Holst J.J. (2017). Improvement of Insulin Sensitivity after Lean Donor Feces in Metabolic Syndrome Is Driven by Baseline Intestinal Microbiota Composition. Cell Metab..

[111] Mocanu V., Zhang Z., Deehan E.C., Kao D.H., Hotte N., Karmali S., Birch D.W., Samarasinghe K.K., Walter J., Madsen K.L. (2021). Fecal microbial transplantation and fiber supplementation in patients with severe obesity and metabolic syndrome: A randomized double-blind, placebo-controlled phase 2 trial. Nat. Med..

[112] Yu E.W., Gao L., Stastka P., Cheney M.C., Mahabamunuge J., Torres Soto M., Ford C.B., Bryant J.A., Henn M.R., Hohmann E.L. (2020). Fecal microbiota transplantation for the improvement of metabolism in obesity: The FMT-TRIM double-blind placebo-controlled pilot trial. PLoS Med..

[113] Zhao J., Duan L., Li J., Yao C., Wang G., Mi J., Yu Y., Ding L., Zhao Y., Yan G. (2024). New insights into the interplay between autophagy, gut microbiota and insulin resistance in metabolic syndrome. Biomed. Pharmacother..

[114] de Groot P.F., Frissen M.N., de Clercq N.C., Nieuwdorp M. (2017). Fecal microbiota transplantation in metabolic syndrome: History, present and future. Gut Microbes.

[115] Backhed F., Ding H., Wang T., Hooper L.V., Koh G.Y., Nagy A., Semenkovich C.F., Gordon J.I. (2004). The gut microbiota as an environmental factor that regulates fat storage. Proc. Natl. Acad. Sci. USA.

[116] Maruvada P., Leone V., Kaplan L.M., Chang E.B. (2017). The Human Microbiome and Obesity: Moving beyond Associations. Cell Host Microbe.

[117] Zeevi D., Korem T., Zmora N., Israeli D., Rothschild D., Weinberger A., Ben-Yacov O., Lador D., Avnit-Sagi T., Lotan-Pompan M. (2015). Personalized Nutrition by Prediction of Glycemic Responses. Cell.

[118] Berry S.E., Valdes A.M., Drew D.A., Asnicar F., Mazidi M., Wolf J., Capdevila J., Hadjigeorgiou G., Davies R., Al Khatib H. (2020). Human postprandial responses to food and potential for precision nutrition. Nat. Med..

[119] Mendes-Soares H., Raveh-Sadka T., Azulay S., Ben-Shlomo Y., Cohen Y., Ofek T., Stevens J., Bachrach D., Kashyap P., Segal L. (2019). Model of personalized postprandial glycemic response to food developed for an Israeli cohort predicts responses in Midwestern American individuals. Am. J. Clin. Nutr..

[120] Ben-Yacov O., Godneva A., Rein M., Shilo S., Lotan-Pompan M., Weinberger A., Segal E. (2023). Gut microbiome modulates the effects of a personalised postprandial-targeting (PPT) diet on cardiometabolic markers: A diet intervention in pre-diabetes. Gut.

[121] Song E.J., Shin J.H. (2022). Personalized Diets based on the Gut Microbiome as a Target for Health Maintenance: From Current Evidence to Future Possibilities. J. Microbiol. Biotechnol..

[122] Popp C.J., Hu L., Kharmats A.Y., Curran M., Berube L., Wang C., Pompeii M.L., Illiano P., St-Jules D.E., Mottern M. (2022). Effect of a Personalized Diet to Reduce Postprandial Glycemic Response vs a Low-fat Diet on Weight Loss in Adults with Abnormal Glucose Metabolism and Obesity: A Randomized Clinical Trial. JAMA Netw. Open.

[123] Sheflin A.M., Melby C.L., Carbonero F., Weir T.L. (2017). Linking dietary patterns with gut microbial composition and function. Gut Microbes.

[124] Koh A., De Vadder F., Kovatcheva-Datchary P., Backhed F. (2016). From Dietary Fiber to Host Physiology: Short-Chain Fatty Acids as Key Bacterial Metabolites. Cell.

[125] Magne F., Gotteland M., Gauthier L., Zazueta A., Pesoa S., Navarrete P., Balamurugan R. (2020). The Firmicutes/Bacteroidetes Ratio: A Relevant Marker of Gut Dysbiosis in Obese Patients?. Nutrients.

[126] Yang S.Y., Han S.M., Lee J.Y., Kim K.S., Lee J.E., Lee D.W. (2025). Advancing Gut Microbiome Research: The Shift from Metagenomics to Multi-Omics and Future Perspectives. J. Microbiol. Biotechnol..

[127] Marcil V., Delvin E., Seidman E., Poitras L., Zoltowska M., Garofalo C., Levy E. (2002). Modulation of lipid synthesis, apolipoprotein biogenesis, and lipoprotein assembly by butyrate. Am. J. Physiol. Gastrointest. Liver Physiol..

[128] El-Kurjieh A., Al-Arab R., Hachem Q.A., Ibrahim J.N., Kobeissy P.H. (2025). ACSS2 and metabolic diseases: From lipid metabolism to therapeutic target. Lipids Health Dis..

[129] Heintz-Buschart A., Wilmes P. (2018). Human Gut Microbiome: Function Matters. Trends Microbiol..

[130] Knight R., Vrbanac A., Taylor B.C., Aksenov A., Callewaert C., Debelius J., Gonzalez A., Kosciolek T., McCall L.I., McDonald D. (2018). Best practices for analysing microbiomes. Nat. Rev. Microbiol..

[131] Yatsunenko T., Rey F.E., Manary M.J., Trehan I., Dominguez-Bello M.G., Contreras M., Magris M., Hidalgo G., Baldassano R.N., Anokhin A.P. (2012). Human gut microbiome viewed across age and geography. Nature.

[132] Falony G., Joossens M., Vieira-Silva S., Wang J., Darzi Y., Faust K., Kurilshikov A., Bonder M.J., Valles-Colomer M., Vandeputte D. (2016). Population-level analysis of gut microbiome variation. Science.

[133] Rothschild D., Weissbrod O., Barkan E., Kurilshikov A., Korem T., Zeevi D., Costea P.I., Godneva A., Kalka I.N., Bar N. (2018). Environment dominates over host genetics in shaping human gut microbiota. Nature.

[134] Vujkovic-Cvijin I., Sklar J., Jiang L., Natarajan L., Knight R., Belkaid Y. (2020). Host variables confound gut microbiota studies of human disease. Nature.

